# Insurance patterns and instability from 2006 to 2016

**DOI:** 10.1186/s12913-020-05226-1

**Published:** 2020-04-21

**Authors:** Yunwei Gai, Kent Jones

**Affiliations:** Economics Division, Babson College, 231 Forest Street, Babson Park, MA 02457-0310 USA

**Keywords:** Insurance instability, Affordable care act, Medical expenditure panel survey, Health insurance disparities, Health care utilization

## Abstract

**Background:**

There is a rich literature on insurance coverage and its impacts on health care. Many recent studies have examined the impacts of the Affordable Care Act (ACA) and found that it had positive effects on health insurance coverage and health care usage. Most of the literature, however, has focused on insurance coverage at a single point in time, while research on insurance instability is underrepresented, even though it could significantly impact health outcomes. The aim of this study is to examine changes and implications of insurance instability among nonelderly adults from 2006 to 2016, covering the Great Recession and post-ACA periods.

**Methods:**

Using 2006-to-2016 Medical Expenditure Panel Survey data, we identify seven insurance patterns and analyze them by race/ethnicity, age, geography, income, and medical conditions. We then use multivariable linear models to analyze the relationship between insurance instability and health care status, access, and utilization. Logistic, Poisson and nonlinear models test the robustness of our results.

**Results:**

The post-ACA period 2015–2016 saw the lowest ever-uninsured rate (25.68% or 67.91 million). The largest decrease in insurance instability was among adults aged 19–25, low-income families, Hispanics, the western population, and the healthy population. Like the always-uninsured, those with other insurance gaps experienced a lack of access to care and decreased preventive care and other services.

**Conclusions:**

Despite the post-ACA instability reduction, over 25% of the U.S. population continued to have insurance gaps over a two-year period. Disparities continued to exist between income groups, race/ethnicities, and regions. Repealing ACA could exacerbate insurance instability and disparities between different groups, which in turn could lead to adverse health outcomes.

## Background

Many studies have examined the impacts of the Affordable Care Act (ACA) and found that it had positive effects on health insurance coverage and health care usage [1–10] through Medicaid expansion, health insurance exchanges, parental coverage extension, individual mandates, cost sharing and subsidies, and employer mandates [11]. Most of the literature has focused on insurance coverage at a single point in time [12, 13] while research on insurance instability is underrepresented [14], even though it could significantly impact health outcomes. For instance, one study found that 89 million people had at least 1 month without insurance between 2004 and 2007 [13], which more than doubled the 43 million uninsured during the sample period based on a point-in-time measure [15]. Insurance interruptions can result in adverse health outcomes similar to those experienced by the continuously uninsured [12–14, 16].

Studies on insurance stability focus on those who are less than 65 years old, since Medicare covers people who are 65 and above. They often use data from the Survey of Income and Program Participation (SIPP) or Medical Expenditure Panel Survey (MEPS) [14]. Both are large-scale panel surveys. SIPP focuses on data related to the income and program participation of individuals and households in the United States. Before the 2014 panel, SIPP interviewed respondents every 4 months over a period of 4 years, asking their monthly insurance status and coverage types in each interview, as well as their demographic and socioeconomic information. These features made SIPP suitable for analyzing insurance stability over a four-year period. Details are at https://www.census.gov/sipp/

MEPS focuses on health status, health services and costs in the United States. Each MEPS panel interviews the same individual and family five times over a two-year period. The survey has three major components: (1) the household component (HC), which is the core survey that collects data on family and individual demographic characteristics, medical expenses, medical conditions, health service use, including ER visits, physician services and prescribed medications, employment status, and health insurance status for each month; (2) the medical provider component (MPC), which collects information from hospitals, doctors, home health care providers, and pharmacies to compare and supplement the information from the HC; and (3) the insurance component (IC), which is an independent survey of employers on the health insurance they provide to their employees. The first MEPS panel (i.e., panel 1) was conducted from 1996 to 1997. This study uses the surveys from 2006 to 2016 (i.e., panel 11 to panel 20). Details are at https://www.meps.ahrq.gov/mepsweb/.

The present study uses MEPS instead of SIPP for the following reasons. First, the most recent SIPP panel covers data only through December 2014, thus excluding the impact of key provisions of the ACA in 2014 and later. In comparison, the most recent MEPS data (i.e., panel 20) covers the period of 2015 to the end of 2016, thus allowing an analysis of changes after the complete ACA roll-out. As in all MEPS data, panel 20 interviewed individuals and families five times from 2015 to 2016.

Second, the earlier SIPP interview pattern created a “seam” problem, as noted in previous studies [12, 13]. Since insurance status was measured in a four-month interval, SIPP respondents tended to report more changes at the beginning of each interview period than within the four-month period. The 2014 panel did away with the four-month interviews, instead of collecting responses once per year, thus a total of four times in the four-year survey period. This could exacerbate the “seam” problem and may lead to less reliable responses. In comparison, MEPS interviews occurred five times during each panel’s two-year period, asking respondents about monthly insurance status in each round, thus at a much higher frequency than SIPP. Responses in MEPS were verified by other sources, including insurance companies, medical providers and employers, and are therefore less likely to suffer from the “seam” and related reporting errors. In addition, unlike SIPP, the longitudinal survey weights in MEPS compensate for panel attrition [12, 17].

Third, MEPS has detailed information on respondents’ health status, medical conditions and health care use, including measures of physical and mental health, ICD-9 codes for diagnoses and procedures, and utilization records such as physician visits, and preventive care services. Such information is important for analyzing both the insurance stability among people with chronic conditions and the relationship between insurance stability and health care services.

While MEPS is limited in its two-year span for each panel, by using data from 2006 to 2016, we conduct a long-term comparison of insurance stability over 11 years, covering two important landmarks: the Great Recession and the passage and roll-out of the 2010 ACA. We analyze these changes in the entire population and sub-population by age, income, race/ethnicity, regions and medical conditions. We also study the relationship between insurance instability and access to care, preventive care, health behavior, health status, expenditure, and utilization. We thus provide a comprehensive picture of health insurance instability, including trends and changes following the Great Recession and the ACA, differences across various population groups, and the impacts of various types of insurance interruptions on health status and outcomes.

## Methods

Data used in this study come from the 2006-to-2016 MEPS (i.e., panels 11 to 20), with the sample restricted to the population below age 65 and those with complete monthly insurance status. The MEPS Panel 11 includes five rounds of interviews of the same individuals and families conducted from 2006 to 2007. Panel 12 surveys another group of individuals and families five times from 2007 to 2008. The other panels follow a similar pattern. The publicly available data has removed all identifying information in order to protect the privacy of individual patients, physicians, and hospitals. An exemption is granted by the Babson College Institutional Review Board. Each MEPS panel includes five rounds of interviews during 2 years and each round contains questions on health status, insurance coverage, health care utilization and expenditure, as well as socioeconomic and demographic information [18].

MEPS panels record respondents’ monthly public and/or private coverage. Private coverage includes employer-provided health insurance (EPHI), self-purchased insurance (such as from the health insurance exchange), and other types of private insurance. Public insurance includes Medicare, Medicaid/SCHIP (State Children’s Health Insurance Programs), TRICARE/CHAMPVA, and other public sources. Like SIPP, MEPS does not separate Medicaid and SCHIP. These categories are not mutually exclusive. Individuals can have multiple coverages (e.g., both EPHI and non-group insurance). Beginning in 2014 “Federal/State Exchange” was added to the list of private insurance categories.

### Insurance patterns

**Fig. 1:**
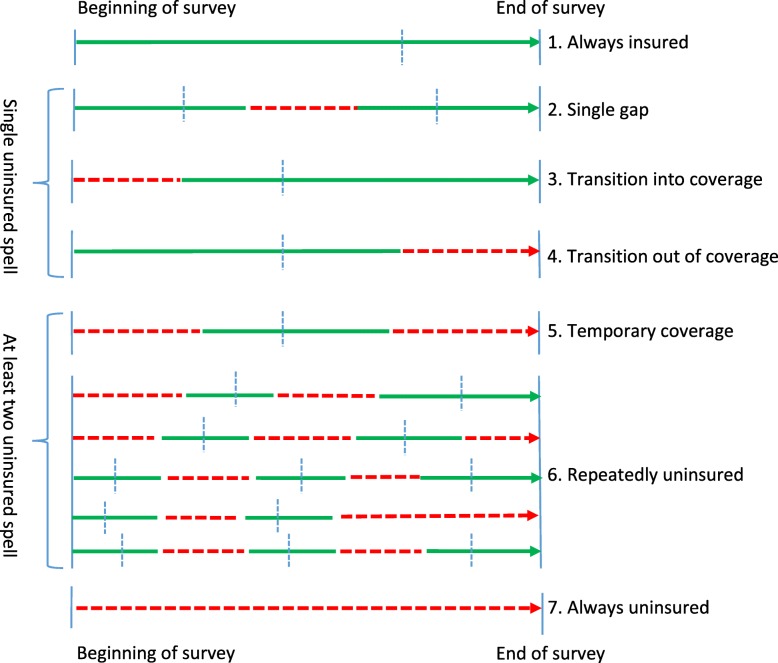
Seven Insurance Patterns. Note: Green lines indicate insured periods; red dashed lines indicate uninsured periods; vertical dashed blue lines indicate uninterrupted transitions between private and public insurance

In this paper, we define seven insurance patterns, consistent with previous studies [12–14]. As shown in Fig. 1, patterns are ordered by the number of uninsured spells: (1) always insured, (2) single gap, (3) transition into coverage, (4) transition out of coverage, (5) temporary coverage, (6) repeatedly uninsured and (7) always uninsured. Patterns 2, 3 and 4 have only one uninsured spell; while patterns 5 and 6 have at least two spells. Within each pattern, except (7), a person could experience switches between private and public insurance, as indicated by the dotted blue line [12, 13].

We analyze each of the seven patterns in terms of their percentage of the total population, their sample-weighted population, the average number of uninsured spells, and the percentages of coverage by private and public sources. We then examine them by three age groups (0–18, 19–25, and 26–64), income groups by the Federal Poverty Line (< 200% FPL, 200 to 399% FPL and 400 + % FPL), geographic regions (Northeast, Midwest, South and West), race/ethnicity (non-Hispanic white, non-Hispanic black, Hispanic, and non-Hispanic other races), and whether a respondent is diagnosed with priority conditions. Priority conditions, as specified by the Agency for Healthcare Research and Quality (AHRQ), include hypertension, heart disease, high cholesterol, emphysema, chronic bronchitis, diabetes, cancer, arthritis, asthma, attention-deficit/hyperactivity disorder, and stroke. AHRQ categorizes them as priority conditions because of their prevalence, expense, relevance to policy and because standards for care for them have been developed [19, 20].

### Dependent variables

After examining patterns of insurance instability, we analyze their relationship with lack of access to care, preventive care, health behavior, health status, and health services utilization. We create a total of 16 measures. Using each one as the dependent variable, we estimate 16 linear models.

A dichotomous variable measures the lack of access to care, equaling one if a respondent answered yes to any of the following questions during the two-year survey: (1) Did you lack a usual source of care if you were sick or needed advice about your health? (2) Were you unable to or delayed in getting necessary medical care? (3) Were you unable to or delayed in getting necessary dental care? and (4) Were you unable to or delayed in getting necessary prescription medicine?

There are two measures of the use of preventive care [21]. The participation measure is a dichotomous indicator of whether an eligible person used any of the following services during the 2 years: routine physical check-up, dental check-up, influenza vaccination, blood pressure check, cholesterol check, prostate specific antigen test, pap smear, breast exam, mammogram, colonoscopy, and sigmoidoscopy. The volume measure counts the total number of these listed services an eligible respondent used during the 2 years.

To measure health behavior, we use two dichotomous indicators on whether a person smoked during the two-year period, and whether a person spent a half hour or more in moderate to vigorous physical activity at least five times a week during the survey.

We measure a person’s physical and mental health by three dichotomous variables and four continuous variables. The dichotomous variables each equal one if a respondent’s perceived health was fair or poor; if perceived mental health was fair or poor; or if they were overweight or obese. The continuous variables include the Body Mass Index for adults aged 18 or older; the Kessler index for non-specific psychological distress; the Physical Component Summary (PCS); and the Mental Component Summary (MCS). The Kessler index is a sum of six mental health-related questions in the MEPS’ Self-Administered Questionnaire; a higher index value indicates a greater tendency towards mental disability. PCS and MCS are generic health status instruments to assess health-related quality of life (HRQoL) with higher scores indicating better HRQoL [22]. All health status measures are based on their last responses in the two-year survey.

Health service utilizations include total health care expenditure ($ thousands), number of office visits, number of dental care visits, and number of prescribed medicines in the two-year period. Total health care expenditure includes out-of-pocket and insurance payments, adjusted by inflation to 2016 price levels.

### Independent variables

Our key independent variables are the seven insurance patterns. In each model, we include six dichotomous variables for each type of insurance instability (patterns 2–7 in Fig. 1), with “always insured” as the comparison category. This allows us to compare how each pattern is associated with the outcome variable and its relative importance in that model. In each model, we control for respondents’ socioeconomic characteristics, demographics, general health, and priority conditions. Socioeconomic characteristics include respondents’ level of education, household income, employment status, and insurance patterns. Categories for education include less than high school, high school, some college, and college/post-baccalaureate education.

We measure household income by two-year average family income ($ thousands), adjusted by inflation to 2016 prices. Employment status for the two-year period is measured by whether the respondent was ever unemployed, and whether the respondent was ever a student, during the period. Demographics include gender, race/ethnicity, age and marital status at the start of the survey, and two-year average family size. Age is divided into six categories: 0–18, 19-to-25, 26-to-35, 36-to-45, 46-to-55, and 56-to-64 years of age. We include panel indicators to account for the effects of trends, and geographic indicators (Northeast, Midwest, South, and West) to account for regional variations.

Except for models focusing on health status, we control respondents’ general health during the survey, grouped into three categories: “excellent/very good,” “good,” and “fair/poor”. For models of the utilization of health services, we also add dichotomous variables for each of the priority conditions.

We use multivariable linear probability models for dichotomous dependent variables, and ordinary least squares (OLS) models for count or continuous dependent variables. We use linear models because they allow coefficients to be directly interpreted as probability or quantity changes, and they are reliable for measuring average effects [6, 7, 10, 21, 23]. Our conclusions are robust by using logit models for dichotomous dependent variables, Poisson models for count variables, and log-linear for continuous dependent variables. We use MEPS’ longitudinal weight to account for its survey design and to create nationally representative results. Stata 15 MP (StataCorp LP, College Station, TX) is used for our analysis.

## Results

Table 1 presents the percentages and standard errors, number of observations in the sample, and sample-weighted populations in each insurance pattern over the 2006–2016 sample period. The Great Recession’s impact is reflected in the decrease of always-insured from 68.33% (or 173.68 million) during 2006–2007 to 65.43% (or 168.89 million) during 2008–2009. Accordingly, there was an increase of insurance instability (i.e., patterns 2–7 as a whole) during this same period from 31.67% (or 80.48 million) to 34.57% (or 89.23 million). After the recovery in 2009, we saw an increase in the always-insured and a decrease in the ever-uninsured.

**Table 1:** Distributions of Insurance Patterns from 2006 to 2016 among Non-elderly Adults (64 Years and Under)

Year	2006–2007	2007–2008	2008–2009	2009–2010	2010–2011	2011–2012	2012–2013	2013–2014	2014–2015	2015–2016
**(1) Always Insured**										
Percentage^a^	68.33	65.88	65.43	68.31	68.77	68.81	67.25	69.08	71.74	74.32
Standard Error^b^	(0.71)	(0.95)	(0.88)	(0.85)	(0.78)	(0.83)	(0.86)	(0.89)	(0.79)	(0.81)
# of obs. in sample ^c^	8768	6588	9502	8746	7712	9892	9403	8980	8833	9749
Pop. (millions)^d^	173.68	168.88	168.89	176.98	179.04	179.63	176.47	182.49	189.95	196.50
**(2) Single Gap**										
Percentage	4.73	5.09	5.21	4.55	4.68	4.37	4.38	5.72	5.26	5.30
Standard Error	(0.28)	(0.33)	(0.35)	(0.30)	(0.30)	(0.28)	(0.34)	(0.41)	(0.41)	(0.46)
# of obs. in sample	719	543	785	653	572	719	699	811	708	735
Pop. (in millions)	12.03	13.04	13.44	11.80	12.18	11.42	11.48	15.10	13.92	14.02
**(3) Transition Into Coverage**										
Percentage	7.03	8.47	7.21	6.01	7.12	6.64	7.52	8.97	8.67	6.43
Standard Error	(0.33)	(0.46)	(0.37)	(0.36)	(0.35)	(0.32)	(0.41)	(0.40)	(0.45)	(0.31)
# of obs. in sample	1057	913	1288	917	946	1166	1257	1443	1391	1106
Pop. (in millions)	17.87	21.71	18.61	15.56	18.55	17.34	19.74	23.70	22.96	17.00
**(4) Transition Out of Coverage**										
Percentage	6.11	5.57	5.93	5.80	5.05	5.35	5.53	3.76	3.98	4.03
Standard Error	(0.36)	(0.30)	(0.30)	(0.34)	(0.31)	(0.30)	(0.30)	(0.23)	(0.30)	(0.26)
# of obs. in sample	904	654	1003	840	702	894	927	626	576	639
Pop. (in millions)	15.52	14.27	15.31	15.03	13.15	13.98	14.50	9.93	10.55	10.65
Year	2006–2007	2007–2008	2008–2009	2009–2010	2010–2011	2011–2012	2012–2013	2013–2014	2014–2015	2015–2016
**(5) Temporary Coverage**										
Percentage	1.18	1.45	1.66	1.17	1.04	1.47	1.38	1.05	1.35	1.42
Standard Error	(0.12)	(0.15)	(0.17)	(0.11)	(0.10)	(0.16)	(0.18)	(0.11)	(0.15)	(0.13)
# of obs. in sample	212	168	301	213	170	252	227	190	223	250
Pop. (in millions)	3.00	3.73	4.29	3.04	2.72	3.85	3.62	2.77	3.57	3.74
**(6) Repeatedly Uninsured**										
Percentage	1.41	1.70	1.81	1.57	1.52	1.36	1.30	1.67	1.55	1.64
Standard Error	(0.16)	(0.16)	(0.16)	(0.16)	(0.17)	(0.14)	(0.15)	(0.16)	(0.14)	(0.17)
# of obs. in sample	267	195	315	236	212	239	249	282	250	260
Pop. (in millions)	3.60	4.37	4.67	4.07	3.95	3.54	3.42	4.41	4.09	4.34
**(7) Always Uninsured**										
Percentage	11.20	11.84	12.75	12.58	11.82	11.98	12.64	9.76	7.45	6.87
Standard Error	(0.44)	(0.60)	(0.53)	(0.59)	(0.48)	(0.50)	(0.59)	(0.51)	(0.40)	(0.41)
# of obs. in sample	1907	1436	2435	2074	1926	2428	2561	1875	1409	1385
Pop. (in millions)	28.47	30.34	32.90	32.59	30.77	31.28	33.17	25.80	19.72	18.16
**(8) Any Insurance Interruption, i.e. Patterns Two to Seven as a Whole**										
Percentage	31.67	34.12	34.57	31.69	31.23	31.19	32.75	30.92	28.26	25.68
Standard Error	(0.71)	(0.95)	(0.88)	(0.85)	(0.78)	(0.83)	(0.86)	(0.89)	(0.79)	(0.81)
# of obs. in sample	5066	3909	6127	4933	4528	5698	5920	5227	4557	4375
Pop. (in millions)	80.48	87.45	89.23	82.09	81.31	81.41	85.93	81.70	74.81	67.91

^a^an insurance pattern (e.g., always insured) as a percentage of the total seven patterns including (1) always insured, (2) single gap, (3) transition into coverage, (4) transition out of coverage, (5) temporary coverage, (6) repeatedly uninsured and (7) always uninsured

^b^Standard Error of the percentage; ^c^Number of observations in the sample; ^d^Sample-weighted population in millions for an insurance pattern

Although the ACA was passed in 2010, major components of the law, such as individual mandates, employer requirements, Medicaid expansion, cost-sharing and subsidies, and health insurance exchanges did not take full effect until 2014 or later [11]. This is reflected in the steady and gradual increase of the always-insured after the 2012–2013 period. The always-insured rate of 74.32% (or 196.50 million) during 2015–2016 was significantly higher than pre-ACA periods of 2006 to 2010, and the ACA implementation periods of 2011 to 2013. During 2015–2016, the ever-uninsured rate (i.e., patterns 2 to 7) of 25.68% (or 67.91 million) was the lowest in the entire sample period.

Table 2 lists the average number of uninsured months for patterns 2–6, i.e. any interruption except “always uninsured”. Compared to the beginning of the sample period (i.e., 2006–2007), there were small declines in uninsured spells in the recent years (i.e., 2015–2016). The largest decrease is 1.78 months in the category of “repeatedly uninsured”, followed by 1.75 months in “transition out of coverage” and 1.60 months in “transition into coverage”. Although the overall trend was downward from 2006 to 2016, there were years when the uninsured months increased and deviated from the downward trend. For example, compared to the year 2012–2013, uninsured spells in the groups of “transition into coverage”, “transition out of coverage” and “temporary coverage” increased in the year 2013–2014. This could be due to the temporary instability when many key provisions of the ACA took effect during 2013 and 2014. During this implementation period, it might take some time (hence the gaps in coverage) for individuals and families to enroll or switch insurance policies. Despite this increase in point estimates, the uninsured months in the year 2013 to 2014 are not significantly different from the previous period of 2012 to 2013.

**Table 2:** Length of Uninsured Spells from 2006 to 2016 among Non-elderly Adults (64 Years and Under)

Year	Single Gap in Coverage	Transition Into Coverage	Transition Out Coverage	Temporary Coverage	Repeatedly Uninsured
2006–2007	5.06^a^ (0.28)^b^	9.29 (0.31)	11.39 (0.37)	15.26 (0.40)	10.11 (0.54)
2007–2008	5.18 (0.24)	10.03 (0.38)	10.32 (0.34)	14.88 (0.45)	10.92 (0.50)
2008–2009	4.77 (0.22)	9.38 (0.35)	10.76 (0.31)	14.95 (0.46)	9.64 (0.39)
2009–2010	5.23 (0.33)	9.18 (0.36)	10.67 (0.38)	15.03 (0.43)	9.26 (0.54)
2010–2011	4.68 (0.25)	9.66 (0.34)	10.26 (0.35)	14.58 (0.44)	9.73 (0.38)
2011–2012	5.02 (0.26)	9.62 (0.26)	10.69 (0.34)	15.01 (0.54)	9.20 (0.37)
2012–2013	5.04 (0.26)	9.74 (0.32)	9.80 (0.37)	14.81 (0.43)	9.91 (0.46)
2013–2014	4.81 (0.19)	10.47 (0.29)	10.89 (0.44)	15.22 (0.56)	9.73 (0.39)
2014–2015	4.28 (0.22)	8.65 (0.32)	9.16 (0.44)	14.02 (0.54)	8.73 (0.40)
2015–2016	4.10 (0.17)	7.69 (0.23)	9.64 (0.40)	13.67 (0.57)	8.33 (0.53)

^a^Uninsured spells are measured in months. ^b^Standard errors are reported in parentheses

MEPS’ longitudinal weights were used to derive the number of months and the standard errors.

In each pattern, a person could have public or private insurance, and sometimes both. Table 3 lists the percentages of public and private insurance the respondents had ever obtained at some point in time during the two-year period. The last column in Table 3 compiles information on all insurance interruptions. There was an increase in public insurance and a decrease in private insurance among people who were always insured, had a single gap or who were ever-uninsured. Medicaid/SCHIP and EPHI accounted for the majority of public and private coverage, respectively. Comparing 2006–2007 with 2015–2016, we observe that, among people who were always insured, the percentages of ever-had public insurance increased from 22.00 to 28.33%, and ever-had private insurance decreased from 84.67 to 80.09%. The percentages of ever-had Medicaid/SCHIP increased from 16.03 to 23.32%; and ever-had EPHI decreased from 79.57 to 71.50%.

**Table 3:** Percentage of Public and Private Insurance from 2006 to 2016 among Non-elderly Adults (64 Years and Under)

Year	Always Insured Pct.^a^(SE)^b^	Single Gap Pct. (SE)	Transition Into Pct. (SE)	Transition Out Pct. (SE)	Temporary Pct. (SE)	Repeatedly Uninsured	Ever Uninsured Pct. (SE)
2006–2007							
Public	22.00^a^ (0.79)^b^	43.09 (2.96)	33.63 (2.32)	30.85 (2.34)	42.07 (4.49)	65.70 (5.48)	37.69 (1.43)
Medicaid^c^	16.03 (0.76)	39.40 (2.89)	28.62 (2.21)	27.23 (2.27)	40.04 (4.43)	61.58 (5.30)	33.64 (1.37)
Private	84.67 (0.74)	76.71 (2.72)	69.76 (2.18)	71.97 (2.23)	60.22 (4.39)	57.49 (5.54)	70.63 (1.37)
Employer^d^	79.57 (0.82)	72.77 (2.82)	61.66 (2.39)	60.94 (2.28)	48.95 (4.85)	53.01 (5.50)	62.68 (1.42)
2007–2008							
Public	23.00 (1.07)	45.97 (3.45)	33.46 (2.20)	33.33 (2.82)	47.16 (5.08)	51.88 (5.18)	38.59 (1.70)
Medicaid	17.66 (0.99)	40.24 (3.44)	28.96 (2.18)	29.84 (2.65)	42.16 (5.04)	47.67 (5.21)	34.05 (1.66)
Private	83.53 (0.97)	81.68 (2.51)	69.63 (2.12)	70.00 (2.79)	53.94 (5.12)	64.37 (4.75)	71.05 (1.57)
Employer	78.67 (1.04)	74.25 (2.71)	55.86 (2.58)	60.07 (3.11)	45.03 (5.03)	57.64 (4.82)	60.54 (1.68)
2008–2009							
Public	22.38 (0.90)	41.88 (3.24)	43.50 (2.30)	25.89 (1.93)	45.99 (4.97)	60.73 (4.14)	39.94 (1.39)
Medicaid	16.87 (0.83)	37.52 (3.15)	37.27 (2.21)	22.14 (1.77)	39.59 (4.80)	55.87 (4.26)	34.94 (1.40)
Private	83.82 (0.79)	80.20 (2.17)	60.84 (2.35)	78.90 (1.61)	56.09 (4.93)	59.99 (4.43)	69.94 (1.32)
Employer	78.99 (0.84)	73.96 (2.54)	45.29 (2.36)	67.87 (2.05)	48.77 (4.90)	55.81 (4.56)	59.41 (1.45)
2009–2010							
Public	23.40 (0.97)	43.81 (3.02)	43.72 (3.06)	29.88 (2.33)	51.46 (5.56)	63.80 (5.18)	41.66 (1.61)
Medicaid	17.64 (0.85)	41.50 (3.03)	37.40 (2.89)	27.77 (2.22)	48.15 (5.28)	55.84 (5.30)	37.63 (1.57)
Private	82.60 (0.87)	75.63 (2.52)	60.24 (2.89)	73.88 (1.99)	50.69 (5.41)	61.14 (5.02)	67.54 (1.47)
Employer	76.98 (0.98)	67.32 (3.07)	47.08 (3.02)	62.88 (2.81)	47.13 (5.48)	53.53 (5.14)	57.23 (1.64)
2010–2011							
Public	25.09 (1.00)	48.26 (3.47)	44.52 (2.47)	36.70 (3.02)	52.89 (4.98)	66.97 (4.75)	45.59 (1.77)
Medicaid	19.33 (0.93)	42.85 (3.57)	37.66 (2.46)	34.11 (2.95)	47.04 (4.93)	64.73 (4.78)	40.61 (1.82)
Private	81.40 (0.90)	74.51 (3.05)	59.71 (2.37)	66.41 (2.82)	48.81 (4.99)	62.84 (4.72)	64.68 (1.59)
Employer	75.88 (1.04)	71.46 (3.12)	47.03 (2.58)	57.25 (2.79)	38.20 (5.06)	56.14 (4.97)	55.81 (1.70)
2011–2012							
Public	26.56 (1.06)	50.69 (3.41)	39.66 (2.32)	33.26 (2.71)	41.88 (4.92)	61.67 (4.50)	42.11 (1.49)
Medicaid	20.27 (0.92)	45.69 (3.45)	32.85 (2.27)	31.50 (2.68)	35.18 (4.86)	58.00 (4.43)	37.36 (1.54)
Private	80.50 (0.85)	74.33 (2.69)	65.83 (2.21)	69.38 (2.60)	60.85 (4.74)	58.81 (4.32)	67.88 (1.38)
Employer	74.97 (0.94)	68.29 (2.87)	50.38 (2.49)	54.54 (2.86)	47.96 (5.48)	55.74 (4.46)	55.81 (1.64)
2012–2013							
Public	26.58 (1.13)	51.09 (3.67)	40.92 (2.54)	36.16 (2.87)	54.41 (4.77)	72.48 (4.69)	44.80 (1.69)
Medicaid	21.22 (1.10)	45.20 (3.70)	34.37 (2.43)	32.72 (2.70)	51.06 (4.98)	66.01 (4.86)	39.47 (1.68)
Private	80.61 (0.95)	71.83 (2.70)	63.80 (2.60)	68.99 (2.79)	45.95 (4.78)	57.49 (4.70)	65.34 (1.68)
Employer	74.91 (1.01)	68.75 (2.86)	49.74 (2.79)	59.06 (2.69)	38.90 (3.90)	55.03 (4.70)	56.04 (1.71)
2013–2014							
Public	27.64 (1.07)	52.72 (3.24)	40.27 (2.42)	39.43 (3.20)	45.78 (5.10)	67.76 (4.09)	45.93 (1.61)
Medicaid	22.07 (0.96)	46.77 (3.05)	34.01 (2.29)	36.30 (3.15)	41.82 (4.80)	57.43 (4.54)	40.10 (1.57)
Private	80.17 (0.94)	75.50 (2.66)	64.81 (2.26)	65.00 (3.26)	54.74 (5.09)	67.05 (4.00)	67.41 (1.58)
Employer	74.16 (0.99)	68.15 (2.89)	49.35 (2.45)	53.49 (3.37)	34.26 (5.00)	56.06 (4.59)	54.95 (1.79)
Exchange^e^	0.93 (0.16)	9.13 (1.85)	8.87 (1.19)	0.45 (0.37)	5.91 (2.66)	8.02 (2.15)	7.23 (0.80)
Public	30.61 (1.09)	49.85 (3.40)	45.96 (2.45)	39.27 (3.81)	46.99 (4.73)	69.41 (5.03)	47.47 (1.94)
Medicaid	23.60 (1.04)	43.73 (3.31)	40.78 (2.29)	36.49 (3.67)	41.52 (4.59)	64.49 (4.98)	42.51 (1.84)
Private	78.80 (0.90)	78.42 (2.50)	62.25 (2.14)	66.84 (3.72)	54.46 (4.80)	60.34 (5.37)	66.57 (1.65)
Employer	71.29 (1.05)	72.21 (2.74)	42.73 (2.44)	51.35 (3.73)	26.37 (4.01)	47.52 (5.07)	51.13 (1.74)
Exchange	2.55 (0.34)	8.48 (1.76)	14.54 (1.74)	1.72 (0.80)	15.20 (4.49)	18.94 (4.68)	10.92 (1.02)
2015–2016							
Public	28.33 (1.05)	49.46 (4.30)	43.93 (2.00)	38.57 (3.06)	44.98 (4.98)	62.50 (5.55)	46.04 (1.72)
Medicaid	23.32 (1.00)	44.84 (4.43)	35.41 (1.90)	36.63 (3.01)	39.17 (4.83)	58.90 (5.61)	40.66 (1.71)
Private	80.09 (0.93)	76.25 (3.03)	64.36 (1.96)	68.85 (2.80)	56.75 (4.90)	67.37 (4.68)	68.36 (1.61)
Employer	71.50 (0.99)	62.72 (3.70)	42.89 (2.25)	48.60 (3.39)	35.65 (4.66)	59.34 (5.49)	50.59 (1.80)
Exchange	4.73 (0.43)	16.94 (3.03)	13.95 (1.63)	7.86 (1.71)	12.61 (3.02)	16.74 (3.87)	13.63 (1.28)

^a^Public or private insurance as a percentage of an insurance pattern. For example, 22% of “always insured” had public insurance; and 16.03 of “always insured” had Medicaid

^b^Standard errors of these percentages in parentheses. MEPS’ longitudinal weights are used to create nationally representative results

^c^Medicaid or SCHIP. MEPS does not separate Medicaid and SCHIP. ^d^Employer-provided/union group insurance

^e^Self-purchased health insurance from Federal or State Health Insurance Exchange

From 2006 to 2016, people who had any insurance interruption increased their percentage of public insurance from 37.69 to 46.04%, Medicaid/SCHIP from 33.64 to 40.66%; and decreased their private insurance from 70.63 to 68.36%, and EPHI from 62.68 to 50.59% (see Table 3, last column). The percentages for health insurance exchanges continued to rise from 7.23% during 2013–2014 to 13.63% during 2015–2016. These observations are consistent with the expansion of Medicaid, and the use of Federal/State Exchange as alternative private insurance to EPHI.

Table 1 in Appendix A lists the summary statistics of demographic and socioeconomic characteristics (age, sex, economic status, educational background, etc.) by insurance patterns. Tables A2 to A17 further examine the insurance instability by age, income, geographic regions, race/ethnicity, and diagnosis with priority conditions. Consistent with the results in Table 1 for the entire population, we generally observe a rise in insurance instability during the Great Recession and decrease in instability after economic recovery and after the passage of the ACA. However, there are disparities across population groups in post-ACA periods.

Among the three age groups, the 19–25 group experienced the largest increase in “always insured” from 44.43% (or 12.69 million) during 2006–2007 to 63.15% (or 19.23 million) during 2015–2016. This age group also experienced the largest decrease in all patterns of insurance instability, which could be the combined effects from the parental coverage extension to young adults [23], individual mandates, employer mandates and other requirements of ACA.

The low-income group, families below 200% FPL, had a larger decrease in insurance instability (49.37 to 39.07%) than both the middle- and high-income groups. However, it is noteworthy that even with the largest decrease in instability, there continued to be a large gap between low-income and other income groups. During 2015–2016, 72.78% of middle-income and 86.29% of high-income groups were continuously insured; compared to only 60.93% of low-income respondents.

Among the four race/ethnicity groups, the Hispanic population had the largest decrease in insurance interruptions of any kind, from 50.94 to 39.04%. However, large gaps continued to exist across race/ethnicity groups. Non-Hispanic whites had the highest percentage of uninterrupted insurance (79.25%) during 2015–2016, followed by non-Hispanic other race/ethnic groups (77.37%), non-Hispanic blacks (70.15%) and Hispanics (60.96%).

The Western region had the largest decrease in insurance instability of any kind, from 35.94% during 2006–2007 to 25.66% during 2015–2016, followed by the Northeast (25.92 to 18.50%), and the South (36.08 to 29.99%). There was little change in insurance instability in the Midwest, despite fluctuations from 2006 to 2016. With 81.50% at the end of the sample period, the Northeast continued to have the highest percentage of uninterrupted insurance among the four regions.

Those with priority conditions increased their rate of uninterrupted coverage from 65.95% during 2006–2007 to 74.67% during 2015–2016. For those without priority conditions, the rate increased from 65.95 to 73.88%. This is likely due to the individual mandate requiring both healthy and sick populations to enroll in insurance.

Table 4 presents the relationships between insurance patterns and lack of access to care, use of preventive care, health behavior, health status, and utilization of health services. All models in Table 4 control for respondents’ socioeconomic characteristics, demographics, general health status, priority conditions, panel indicators, and geographic indicators. The section of “Independent Variables” has a full list of the variables. To be concise, we report only the coefficients for instability in each model, with “always insured” as the comparison group. Full results are available from the authors upon request. Compared to “always insured”, each type of insurance interruption was associated with an increased probability of reduced access to care. The largest increased probability was in “always uninsured” (36%), followed by “temporary coverage” (31%) and “repeatedly uninsured” (27%). Coverage instability was associated with decreases in the probability and number of preventive services, with the largest decrease among the always-uninsured, followed by those with temporary coverage, and those making the transition into or out of health insurance. Those with insurance interruptions were 4–9% more likely to smoke, but their physical activity was similar to or slightly higher than people who were always insured.

**Table 4:** Relationships of Insurance Patterns with Health Care Usage, Status and Outcomes

	Lack of Access	Any Preventive Care	# of Preventive Care	Smoking	Physical Activity	Perceived Poor Health	Perceived Poor Mental Health	Overweight or Obese
Single Gap	0.17***	−0.00	− 0.09***	0.04***	0.02*	−0.01***	− 0.01***	− 0.00
	(0.01)	(0.00)	(0.03)	(0.01)	(0.01)	(0.00)	(0.00)	(0.01)
Transition Into	0.23***	−0.02***	−0.40***	0.05***	0.02***	−0.01*	−0.01***	− 0.02**
	(0.01)	(0.00)	(0.02)	(0.01)	(0.01)	(0.00)	(0.00)	(0.01)
Transition Out	0.22***	−0.03***	− 0.39***	0.06***	0.02*	−0.03***	− 0.02***	− 0.01
	(0.01)	(0.00)	(0.03)	(0.01)	(0.01)	(0.00)	(0.00)	(0.01)
Temporary Coverage	0.31***	−0.04***	− 0.55***	0.08***	− 0.01	− 0.03***	− 0.03***	−0.02
	(0.01)	(0.01)	(0.06)	(0.01)	(0.01)	(0.01)	(0.01)	(0.01)
Repeatedly Uninsured	0.27***	−0.02***	−0.31***	0.09***	0.02	−0.02***	−0.02***	0.03**
	(0.02)	(0.01)	(0.04)	(0.02)	(0.01)	(0.01)	(0.01)	(0.01)
Always Uninsured	0.36***	−0.11***	−1.32***	0.07***	0.02***	−0.03***	−0.03***	− 0.04***
	(0.01)	(0.00)	(0.03)	(0.01)	(0.01)	(0.00)	(0.00)	(0.01)
# of obs.	123,324	123,148	123,331	91,064	94,703	123,331	123,331	94,181
R^2^	0.20	0.07	0.64	0.14	0.06	0.13	0.07	0.12
	**Kessler Index**	**Physical Comp. Summary**	**Mental Comp. Summary**	**BMI**	**Total Health Expenditure**	**# of Office Visits**	**# of Dental Visits**	**# of Prescriptions**
Single Gap	0.02	1.02***	−0.18	−0.23*	−0.13	−2.32***	− 0.32	−2.27*
	(0.08)	(0.16)	(0.19)	(0.13)	(1.16)	(0.71)	(0.33)	(1.30)
Transition Into	0.06	0.47***	−0.21	−0.49***	−0.68	−2.16***	− 0.84***	− 2.34**
	(0.06)	(0.12)	(0.14)	(0.10)	(0.62)	(0.57)	(0.17)	(1.03)
Transition Out	−0.11	1.21***	−0.05	− 0.38***	−1.60***	−1.95***	−0.67***	−1.62**
	(0.07)	(0.14)	(0.16)	(0.12)	(0.41)	(0.53)	(0.20)	(0.74)
Temporary Coverage	0.10	1.21***	−0.38	−0.32	−1.11*	−2.92***	− 1.04***	−3.70***
	(0.14)	(0.25)	(0.30)	(0.20)	(0.61)	(0.57)	(0.22)	(0.79)
Repeatedly Uninsured	0.30**	1.22***	−0.51	0.20	−2.31***	−2.00***	− 0.94***	−3.74***
	(0.13)	(0.28)	(0.31)	(0.21)	(0.86)	(0.74)	(0.31)	(0.86)
Always Uninsured	−0.19***	1.41***	0.14	−0.87***	−1.91***	−1.83*	−1.11***	−3.29***
	(0.06)	(0.11)	(0.11)	(0.09)	(0.42)	(1.08)	(0.16)	(0.57)
# of obs.	90,820	91,230	91,232	137,660	3706	3706	3706	3706
R^2^	0.13	0.25	0.10	0.11	0.22	0.19	0.10	0.26

*p < 0.10; **p < 0.05; ***p < 0.01

Standard errors are reported in parentheses. MEPS’s longitudinal sample weights are applied in each regression

All models included respondents’ socioeconomic characteristics, demographics, general health status, priority conditions, panel indicators, and geographic indicators. Please refer to “independent variables” section for a full list of the variables

For conciseness, these coefficients are not reported here. Full results are available from the authors on request

With a few exceptions, people with unstable coverage were at least as healthy as those with continuous coverage in all measures of health status, based on the self-perceived health measures and indexes described earlier. This could be due to the general observation that healthier people are less likely to purchase health insurance. For example, compared to “always insured”, a single gap was associated with a 1.22% less probability of poor health and “repeatedly uninsured” was associated with a 1.87% less probability. Because both insurance patterns were compared to “always insured”, we can infer that “repeatedly uninsured” was associated with 1.87–1.22% = 0.65% less probability of poor health than “single gap”. Furthermore, the difference is statistically significant between “repeatedly uninsured” and “single gap” because there is no overlap between the 95% confidence intervals of the two variables (− 0.0122 ± 0.0037 × 1.96 = (− 0.0195, − 0.0049) for single gap; and − 0.0187 ± 0.007 × 1.96 = (− 0.032, − 0.0056) for repeatedly uninsured). The other pairwise comparisons between insurance patterns can be derived similarly from Table 4.

Insurance instability was associated with less utilization of health services, including total health expenditure, office, and dental visits, and number of prescribed medicines. With the exception of dental visits, the largest decrease in health care utilization was not among people who were always uninsured, but rather those who were repeatedly uninsured, had temporary coverage or had a single gap. However, the differences among these insurance instabilities were not statistically significant.

Appendix B reports results from logit, Poisson, and log-linear models as robustness tests, which support our results. In another robustness test, we created a dummy variable that equaled one if there were transitions between private and public insurance. We then included it as an additional independent variable in all models. As shown in Table B2 in the appendix, the changes in the coefficients for insurance patterns were small and comparable to the results in Table 4. Therefore, our conclusions remain the same.

In Table B3, we replaced insurance interruption indicators with the number of months a person was uninsured to measure the dose response, i.e., how the spell of non-coverage affects the health status and other outcomes. For the ease of interpretation, we use multivariable linear models for our analyses. As shown in the table, every additional month of non-coverage is associated with a 1.55% increase in the probability of reduced access to care, and a 0.415% decrease in the probability of using preventive care. The longer the insurance interruptions, the worse outcome in accessing and utilizing health care resources. Each additional month of non-coverage is associated with increased health. As discussed previously, this could be due to the moral hazard problem, reverse causality and omitted variable bias. Overall, the results are consistent with the main findings and conclusions in the paper.

## Discussion

Analysis of insurance interruptions is underrepresented in the literature [14], even though studies have shown that insurance instability could lead to discontinuity of care, increased financial risks and adverse health outcomes [12–14]. Using 2006–2016 MEPS data, this study contributes to the literature and public health policy by providing a comprehensive analysis of insurance instability during this period and its relationship to health status, health outcomes and health care utilization. Important economic and policy events, such as the Great Recession and the ACA, clearly affected insurance instabilities.

The ACA decreased both insurance instability and uninsured spells. The largest decreases occurred among the ACA’s intended population groups: adults aged 19–25, low-income families, and minority populations. There was increased reliance on public health insurance, particularly Medicaid, and a decrease in EPHI, coupled with an increase in health insurance exchanges as alternative private insurance. Yet, despite significant post-ACA improvement in insurance stability, the data show that over 25% of the U. S. population still experienced at least one uninsured spell in a given two-year period, and disparities continue to exist across population groups and regions.

The low-income group, Hispanic population, and the Northeast region had larger decreases in insurance instability than other income groups, race/ethnicity groups and regions. This difference is likely due to the Medicaid expansion under the 2010 Affordable Care Act, which extended Medicaid eligibility to families with income below 138% of the federal poverty level. There is a higher percentage of eligible families in the Hispanic population than in other race/ethnicity groups. We, therefore, observe larger decreases in insurance gaps among low-income families and the Hispanic population. Most of the states in the Northeast region have expanded Medicaid, while other regions, especially the South and Midwest, have not. Some states in the Northeast, such as Massachusetts and New York State, also have their own public health insurance programs to provide coverage. Therefore, the Northeast continued to have the highest percentage of uninterrupted insurance among the four regions. Based on these observations, future research should include an in-depth study to examine how government subsidies, public insurance programs, and other policies can be adjusted to reduce the disparities and further improve insurance stability across population groups and regions.

As with the always-uninsured group, people with other kinds of instability experienced a lack of access to care and reduced health services. In some cases, those with temporary coverage and repeated non-coverage suffered equal or larger negative impacts on health care than those who were continuously uninsured. Although we observe an increased probability of reduced access to care and a decrease in the use of preventive services among people with insurance gaps, it is unclear how and in what way they could affect people’s health status and health behavior. For instance, people with insurance gaps had similar or slightly higher physical activity compared to people with continuous insurance. They were also as healthy as those with continuous coverage in all measures of health status. One explanation is that because of the lack of insurance, they could be trying to stay healthy and prevent health problems through physical exercise; while insured people could be less concerned about their health.

This speculation is consistent with the moral hazard problem. But there could also be other explanations such as reverse causality and omitted variable bias. Insured people could have more health problems, which in turn cause them to use more health services while preventing them from engaging in physical activities (e.g., respiratory illnesses). Although we control for people’s demographic and socioeconomic factors, there could be omitted variables (e.g., history of family health, genetic disposition, and environmental factors) that could explain these findings and relationships. Analyzing the causality relationship will require data with external shocks to each of the factors in the model and econometric methods such as the use of instrumental variable or difference-in-difference design. The MEPS data do not have such external shocks; therefore these methods would be outside the scope of this paper.

Our study has some limitations. First, MEPS uses two-year panels compared to SIPP’s four-year panels; findings in this paper are therefore not directly comparable with studies using SIPP. However, our findings on the ever-uninsured population of 80.48 million during 2006–2007 and 87.45 million during 2007–2008 are close to the 89.00 million from the 2004–2007 SIPP [13]. Because of the shorter panels we are likely to underestimate the ever-uninsured population, and our findings could be interpreted as the lower bound of insurance instability. Nonetheless, our study presents strong evidence regarding the changes during the sample period.

Second, small sample sizes could lead to unreliable estimates, as with the fewer than ten observations for the “Non-Hispanic other races” group in the temporary coverage insurance pattern. By comparison, instability of any kind had larger sample sizes, thus providing a more reliable estimate of insurance interruption patterns (2) to (7) in Fig. 1 as a group.

Third, our analysis is based on association, not causality. Although we provide some possible explanations, such as moral hazard, to explain the positive relationship between insurance instability and health status, further analysis is needed to examine the causality relationship. For the same reason, it is important to continue research to explain disparities across populations and regions.

Finally, although we could identify who made transitions between private and public insurance, the MEPS data did not include information on the extent of these coverages. For example, a person could transition from a private insurance with limited coverage (or full coverage) to a public insurance with full coverage (or limited coverage); and vice versa. We, therefore, cannot infer which type of insurance was better at influencing health outcomes. A promising and important research stream for the future would be to use more detailed insurance coverage data to analyze this issue.

## Conclusion

Our results provide empirical evidence that insurance instability is an important issue and that the ACA has improved insurance continuity, even though interruptions in coverage continue for a significant portion of the population. Our study provides a comprehensive analysis of this issue at the national level. Realizing its importance, many states and the Federal government have focused attention on the continuity of coverage [14]. Repealing ACA without adequate replacement legislation could negate the progress made so far and exacerbate the problem of insurance instability and disparities between different population groups and regions, which could lead to adverse health outcomes.

## Supplementary information

**Additional file 1.** Appendix A: insurance patterns by three age groups (0–18, 19–25, and 26–64), three income groups by the Federal Poverty Line (less than 200% FPL, 200 to 399% FPL and 400 + % FPL), four geographic regions (Northeast, Midwest, South and West), four race/ethnicity groups (non-Hispanic white, non-Hispanic black, Hispanic, and non-Hispanic other race), and whether a respondent is diagnosed with priority conditions

**Additional file 2.** Appendix B: robustness tests using logit models for dichotomous dependent variables, Poisson models for count variables and log-linear models for continuous dependent variables

## Data Availability

The datasets generated and analyzed during the current study are publicly available in the Medical Expenditure Panel Survey (MEPS): https://meps.ahrq.gov/mepsweb/. MEPS is a set of large-scale surveys of families and individuals, their medical providers, and employers across the United States.

## Abbreviations

ACA: Patient Protection and Affordable Care Act
AHRQ: Agency for Healthcare Research and Quality
EPHI: Employer-provided health insurance
HRQoL: Health-related quality of life
MCS: Mental Component Summary
MEPS: Medical Expenditure Panel Survey
OLS: Ordinary least squares
PCS: Physical Component Summary
SIPP: Survey of Income and Program Participation
SCHIP: State Children’s Health Insurance Program
