# Supplementary material for: Insurance patterns and instability from 2006 to 2016
Source: BMC Health Serv Res. 2020 Apr 21;20:334. doi: 10.1186/s12913-020-05226-1 (PMC7171789; doi:10.1186/s12913-020-05226-1)
Supplement: Supplementary file 1 — Additional file 1. Appendix A: insurance patterns by three age groups (0–18, 19–25, and 26–64), three income groups by the Federal Poverty Line (less than 200% FPL, 200 to 399% FPL and 400 + % FPL), four geographic regions (Northeast, Midwest, South and West), four race/ethnicity groups (non-Hispanic white, non-Hispanic black, Hispanic, and non-Hispanic other race), and whether a respondent is diagnosed with priority conditions [file 12913_2020_5226_MOESM1_ESM.docx]

**Appendix A**

Table 1 in Appendix A lists the summary statistics of demographic and socioeconomic characteristics (age, sex, economic status, educational background, etc.) by insurance patterns. Tables A2 to A17 further examine the insurance pattern by three age groups (0-18, 19-25, and 26-64), three income groups by the Federal Poverty Line (less than 200% FPL, 200% to 399% FPL and 400+% FPL), four geographic regions (Northeast, Midwest, South and West), four race/ethnicity groups (non-Hispanic white, non-Hispanic black, Hispanic, and non-Hispanic other race), and whether a respondent is diagnosed with priority conditions.

| **Table A1. Summary Statistics of Demographics and Socioeconomics by Insurance Patterns** | | | | | | | |
| --- | --- | --- | --- | --- | --- | --- | --- |
|  | Always Insured | Single Gap | Transition Into | Transition Out | Temporary Coverage | Repeatedly Uninsured | Always Uninsured |
| Hispanic | 25.38^a^ | 31.65 | 38.44 | 34.71 | 44.61 | 39.64 | 54.13 |
|  | (0.15)^b^ | (0.56) | (0.45) | (0.54) | (1.06) | (0.98) | (0.36) |
|  |  |  |  |  |  |  |  |
| Non-Hispanic White | 43.86 | 38.62 | 30.59 | 34.13 | 28.65 | 31.46 | 23.60 |
|  | (0.17) | (0.58) | (0.43) | (0.54) | (0.96) | (0.93) | (0.30) |
|  |  |  |  |  |  |  |  |
| Non-Hispanic Black | 19.94 | 20.85 | 22.35 | 22.70 | 20.04 | 21.60 | 15.98 |
|  | (0.13) | (0.49) | (0.39) | (0.48) | (0.85) | (0.82) | (0.26) |
|  |  |  |  |  |  |  |  |
| Non-Hispanic Other | 10.81 | 8.87 | 8.62 | 8.46 | 6.71 | 7.31 | 6.29 |
|  | (0.10) | (0.34) | (0.26) | (0.32) | (0.53) | (0.52) | (0.17) |
|  |  |  |  |  |  |  |  |
| Male | 47.36 | 44.46 | 47.01 | 48.16 | 44.70 | 41.64 | 54.19 |
|  | (0.17) | (0.60) | (0.47) | (0.57) | (1.06) | (0.99) | (0.36) |
|  |  |  |  |  |  |  |  |
| Female | 52.64 | 55.54 | 52.99 | 51.84 | 55.30 | 58.36 | 45.81 |
|  | (0.17) | (0.60) | (0.47) | (0.57) | (1.06) | (0.99) | (0.36) |
|  |  |  |  |  |  |  |  |
| Age 18 and under | 38.85 | 41.88 | 27.34 | 28.63 | 20.49 | 35.65 | 10.79 |
|  | (0.16) | (0.59) | (0.42) | (0.51) | (0.86) | (0.96) | (0.22) |
|  |  |  |  |  |  |  |  |
| Age 19 to 25 | 6.89 | 12.96 | 15.46 | 19.64 | 22.67 | 17.88 | 16.54 |
|  | (0.09) | (0.40) | (0.34) | (0.45) | (0.89) | (0.77) | (0.27) |
|  |  |  |  |  |  |  |  |
| Age 26 to 35 | 12.18 | 16.86 | 18.21 | 17.12 | 22.85 | 18.76 | 24.28 |
|  | (0.11) | (0.45) | (0.36) | (0.43) | (0.89) | (0.78) | (0.31) |
|  |  |  |  |  |  |  |  |
| Age 36 to 45 | 13.92 | 11.74 | 14.12 | 14.51 | 17.27 | 13.01 | 21.39 |
|  | (0.12) | (0.39) | (0.33) | (0.40) | (0.80) | (0.67) | (0.29) |
|  |  |  |  |  |  |  |  |
| Age 46 to 55 | 15.88 | 9.97 | 14.36 | 12.41 | 10.88 | 9.50 | 17.71 |
|  | (0.12) | (0.36) | (0.33) | (0.37) | (0.66) | (0.59) | (0.27) |
|  |  |  |  |  |  |  |  |
| Age 56 to 64 | 12.27 | 6.60 | 10.51 | 7.69 | 5.85 | 5.19 | 9.28 |
|  | (0.11) | (0.30) | (0.29) | (0.30) | (0.50) | (0.44) | (0.21) |
|  |  |  |  |  |  |  |  |
| Married | 35.73 | 26.14 | 30.79 | 28.65 | 29.87 | 24.07 | 38.27 |
|  | (0.16) | (0.53) | (0.43) | (0.51) | (0.97) | (0.85) | (0.35) |
|  |  |  |  |  |  |  |  |
| Less than high schl. | 40.00 | 43.73 | 38.95 | 40.59 | 41.50 | 43.84 | 43.98 |
|  | (0.17) | (0.64) | (0.47) | (0.57) | (1.08) | (1.05) | (0.36) |
|  |  |  |  |  |  |  |  |
| High school | 19.35 | 20.24 | 27.38 | 26.49 | 30.51 | 25.38 | 31.15 |
|  | (0.14) | (0.52) | (0.43) | (0.52) | (1.00) | (0.92) | (0.34) |
|  |  |  |  |  |  |  |  |
| Some college | 18.86 | 20.72 | 21.95 | 21.66 | 20.66 | 21.32 | 17.33 |
|  | (0.14) | (0.52) | (0.40) | (0.48) | (0.88) | (0.87) | (0.27) |
|  |  |  |  |  |  |  |  |
| College and higher | 21.78 | 15.30 | 11.73 | 11.27 | 7.33 | 9.46 | 7.54 |
|  | (0.15) | (0.46) | (0.31) | (0.37) | (0.57) | (0.62) | (0.19) |
|  |  |  |  |  |  |  |  |
| Employed | 41.14 | 27.42 | 36.38 | 28.62 | 30.83 | 25.71 | 44.18 |
|  | (0.17) | (0.54) | (0.45) | (0.51) | (0.98) | (0.87) | (0.36) |
|  |  |  |  |  |  |  |  |
| Student dent | 9.16 | 12.64 | 11.22 | 18.40 | 14.96 | 14.81 | 9.63 |
|  | (0.10) | (0.40) | (0.29) | (0.44) | (0.76) | (0.71) | (0.21) |
|  |  |  |  |  |  |  |  |
| Northeast | 17.01 | 14.13 | 15.09 | 12.50 | 11.92 | 11.78 | 10.14 |
|  | (0.13) | (0.42) | (0.33) | (0.38) | (0.69) | (0.64) | (0.22) |
|  |  |  |  |  |  |  |  |
| Midwest | 20.92 | 21.00 | 15.19 | 17.40 | 15.23 | 18.76 | 12.86 |
|  | (0.14) | (0.49) | (0.33) | (0.43) | (0.77) | (0.78) | (0.24) |
|  |  |  |  |  |  |  |  |
| South | 34.52 | 36.22 | 38.61 | 42.10 | 45.97 | 41.32 | 48.38 |
|  | (0.16) | (0.58) | (0.45) | (0.56) | (1.06) | (0.98) | (0.36) |
|  |  |  |  |  |  |  |  |
| West | 27.54 | 28.66 | 31.11 | 28.00 | 26.88 | 28.14 | 28.62 |
|  | (0.15) | (0.54) | (0.43) | (0.51) | (0.94) | (0.90) | (0.32) |
|  |  |  |  |  |  |  |  |
| Family inc. ($000) | 74.43^c^ | 52.17 | 48.94 | 50.80 | 39.84 | 41.21 | 42.82 |
|  | (0.21) | (0.53) | (0.39) | (0.50) | (0.74) | (0.67) | (0.27) |
|  |  |  |  |  |  |  |  |
| Family size | 3.64^c^ | 3.65 | 3.56 | 3.67 | 3.56 | 3.61 | 3.75 |
|  | (0.01) | (0.02) | (0.02) | (0.02) | (0.04) | (0.04) | (0.01) |
|  |  |  |  |  |  |  |  |
| N | 88173 | 6944 | 11484 | 7765 | 2206 | 2505 | 19436 |
| ^a^Percentages of each category; ^b^standard errors of the percentages.  ^c^Averages of family income (in thousands of dollars) and family size are reported. | | | | | | | |

| **Table A2 Distributions of Insurance Patterns from 2006 to 2016, Age 18 and Below** | | | | | | | | | | |
| --- | --- | --- | --- | --- | --- | --- | --- | --- | --- | --- |
| Year | 2006-2007 | 2007-2008 | 2008-2009 | 2009-2010 | 2010-2011 | 2011-2012 | 2012-2013 | 2013-2014 | 2014-2015 | 2015-2016 |
|  | (1) Always Insured | | | | | | | | | |
| Pct^a^ | 74.34 | 71.95 | 72.32 | 76.85 | 77.48 | 79.86 | 78.68 | 79.93 | 80.86 | 80.81 |
| SE^b^ | (1.11) | (1.38) | (1.20) | (1.15) | (1.11) | (1.00) | (1.14) | (1.14) | (1.19) | (1.23) |
| n^c^ | 3317 | 2434 | 3752 | 3322 | 2997 | 3941 | 3804 | 3663 | 3403 | 3621 |
| N^d^ | 54.85 | 53.56 | 54.06 | 57.51 | 57.79 | 59.32 | 59.10 | 60.09 | 60.51 | 60.08 |
|  | (2) Single Gap | | | | | | | | | |
| Pct | 6.21 | 6.29 | 6.56 | 5.38 | 5.77 | 5.39 | 5.48 | 6.62 | 5.89 | 5.94 |
| SE | (0.55) | (0.66) | (0.62) | (0.58) | (0.61) | (0.49) | (0.59) | (0.84) | (0.73) | (0.83) |
| n | 346 | 244 | 351 | 282 | 254 | 299 | 293 | 318 | 268 | 253 |
| N | 4.58 | 4.69 | 4.90 | 4.02 | 4.31 | 4.01 | 4.11 | 4.97 | 4.41 | 4.42 |
|  | (3) Transition Into Coverage | | | | | | | | | |
| Pct | 5.96 | 8.39 | 7.97 | 5.66 | 5.42 | 4.70 | 5.65 | 5.04 | 4.78 | 4.48 |
| SE | (0.51) | (0.78) | (0.68) | (0.55) | (0.51) | (0.46) | (0.65) | (0.48) | (0.61) | (0.42) |
| n | 336 | 308 | 492 | 296 | 275 | 304 | 324 | 288 | 267 | 250 |
| N | 4.40 | 6.25 | 5.96 | 4.24 | 4.04 | 3.49 | 4.24 | 3.79 | 3.57 | 3.33 |
|  | (4) Transition Out of Coverage | | | | | | | | | |
| Pct | 5.90 | 5.07 | 4.14 | 4.71 | 4.50 | 4.32 | 4.63 | 3.09 | 4.23 | 3.62 |
| SE | (0.64) | (0.52) | (0.45) | (0.51) | (0.51) | (0.47) | (0.50) | (0.34) | (0.53) | (0.45) |
| n | 305 | 202 | 246 | 203 | 209 | 238 | 268 | 173 | 199 | 180 |
| N | 4.35 | 3.77 | 3.09 | 3.53 | 3.36 | 3.21 | 3.48 | 2.32 | 3.16 | 2.69 |
|  | (5) Temporary Coverage | | | | | | | | | |
| Pct | 0.99 | 1.15 | 1.36 | 0.70 | 0.45 | 0.96 | 0.90 | 0.62 | 0.81 | 0.94 |
| SE | (0.19) | (0.25) | (0.29) | (0.17) | (0.12) | (0.24) | (0.30) | (0.16) | (0.21) | (0.24) |
| n | 70 | 41 | 72 | 45 | 25 | 43 | 43 | 34 | 38 | 41 |
| N | .73 | .85 | 1.02 | .52 | .34 | .72 | .68 | .46 | .61 | .70 |
|  | (6) Repeatedly Uninsured | | | | | | | | | |
| Pct | 1.87 | 1.57 | 1.96 | 1.86 | 1.72 | 1.27 | 1.20 | 1.69 | 0.96 | 1.56 |
| SE | (0.32) | (0.33) | (0.25) | (0.33) | (0.36) | (0.23) | (0.30) | (0.30) | (0.17) | (0.32) |
| n | 130 | 77 | 131 | 93 | 76 | 75 | 83 | 89 | 64 | 75 |
| N | 1.38 | 1.17 | 1.46 | 1.39 | 1.28 | .94 | .90 | 1.27 | .71 | 1.16 |
|  | (7) Always Uninsured | | | | | | | | | |
| Pct | 4.73 | 5.58 | 5.70 | 4.83 | 4.66 | 3.50 | 3.47 | 3.03 | 2.48 | 2.64 |
| SE | (0.52) | (0.79) | (0.71) | (0.54) | (0.53) | (0.43) | (0.41) | (0.45) | (0.40) | (0.54) |
| n | 273 | 234 | 304 | 246 | 220 | 189 | 215 | 162 | 127 | 128 |
| N | 3.49 | 4.15 | 4.26 | 3.62 | 3.48 | 2.60 | 2.61 | 2.28 | 1.85 | 1.96 |
|  | (8) Any Insurance Interruption, i.e. Patterns Two to Seven | | | | | | | | | |
| Pct | 25.66 | 28.05 | 27.68 | 23.15 | 22.52 | 20.14 | 21.32 | 20.07 | 19.14 | 19.19 |
| SE | (1.11) | (1.38) | (1.20) | (1.15) | (1.11) | (1.00) | (1.14) | (1.14) | (1.19) | (1.23) |
| n | 1460 | 1106 | 1596 | 1165 | 1059 | 1148 | 1226 | 1064 | 963 | 927 |
| N | 18.94 | 20.88 | 20.70 | 17.32 | 16.80 | 14.96 | 16.02 | 15.09 | 14.33 | 14.27 |
| ^a^Percentage of a pattern in the total seven patterns; ^b^Standard Error of the percentage;  ^c^Number of observations in the sample; ^d^Sample-weighted population in millions for a pattern. | | | | | | | | | | |

| **Table A3 Distributions of Insurance Patterns from 2006 to 2016, Age 19 to 25** | | | | | | | | | | |
| --- | --- | --- | --- | --- | --- | --- | --- | --- | --- | --- |
| Year | 2006-2007 | 2007-2008 | 2008-2009 | 2009-2010 | 2010-2011 | 2011-2012 | 2012-2013 | 2013-2014 | 2014-2015 | 2015-2016 |
|  | (1) Always Insured | | | | | | | | | |
| Pct^a^ | 44.43 | 40.15 | 40.63 | 46.18 | 50.36 | 51.59 | 45.89 | 53.42 | 58.40 | 63.15 |
| SE^b^ | (1.69) | (2.11) | (1.84) | (1.94) | (2.22) | (1.80) | (1.95) | (2.00) | (1.99) | (1.85) |
| n^c^ | 479 | 383 | 598 | 546 | 529 | 731 | 653 | 696 | 680 | 778 |
| N^d^ | 12.69 | 10.70 | 11.70 | 13.47 | 14.55 | 15.69 | 13.47 | 15.43 | 17.52 | 19.23 |
|  | (2) Single Gap | | | | | | | | | |
| Pct | 7.63 | 6.89 | 7.88 | 6.57 | 4.41 | 5.94 | 5.93 | 6.47 | 7.56 | 6.52 |
| SE | (0.86) | (1.14) | (0.84) | (0.91) | (0.74) | (0.83) | (0.91) | (0.84) | (1.01) | (0.84) |
| n | 91 | 61 | 115 | 84 | 53 | 104 | 98 | 107 | 92 | 95 |
| N | 2.18 | 1.84 | 2.27 | 1.92 | 1.28 | 1.81 | 1.74 | 1.87 | 2.27 | 1.99 |
|  | (3) Transition Into Coverage | | | | | | | | | |
| Pct | 10.43 | 13.36 | 10.56 | 8.46 | 12.51 | 11.38 | 11.95 | 13.36 | 11.95 | 8.03 |
| SE | (0.98) | (1.22) | (0.99) | (1.20) | (1.23) | (1.11) | (1.15) | (1.21) | (1.10) | (0.84) |
| n | 138 | 133 | 203 | 130 | 159 | 208 | 212 | 225 | 213 | 154 |
| N | 2.98 | 3.56 | 3.04 | 2.47 | 3.61 | 3.46 | 3.51 | 3.86 | 3.58 | 2.44 |
|  | (4) Transition Out of Coverage | | | | | | | | | |
| Pct | 12.77 | 11.07 | 12.39 | 10.91 | 8.05 | 9.41 | 9.89 | 6.75 | 7.10 | 8.45 |
| SE | (1.20) | (1.13) | (1.00) | (0.96) | (0.93) | (1.00) | (0.99) | (0.80) | (0.97) | (1.05) |
| n | 178 | 121 | 214 | 170 | 117 | 190 | 176 | 123 | 103 | 133 |
| N | 3.64 | 2.95 | 3.57 | 3.18 | 2.33 | 2.86 | 2.90 | 1.95 | 2.13 | 2.57 |
|  | (5) Temporary Coverage | | | | | | | | | |
| Pct | 2.78 | 3.07 | 4.39 | 2.57 | 2.42 | 3.02 | 3.66 | 2.71 | 2.63 | 2.31 |
| SE | (0.50) | (0.66) | (0.74) | (0.43) | (0.47) | (0.44) | (0.82) | (0.51) | (0.54) | (0.43) |
| n | 42 | 31 | 82 | 49 | 38 | 63 | 56 | 52 | 40 | 47 |
| N | .79 | .82 | 1.26 | .75 | .70 | .92 | 1.08 | .78 | .79 | .70 |
|  | (6) Repeatedly Uninsured | | | | | | | | | |
| Pct | 2.42 | 3.40 | 3.54 | 3.10 | 3.07 | 2.20 | 2.80 | 3.45 | 1.47 | 2.29 |
| SE | (0.51) | (0.75) | (0.58) | (0.59) | (0.62) | (0.41) | (0.55) | (0.62) | (0.31) | (0.44) |
| n | 36 | 36 | 59 | 47 | 37 | 52 | 48 | 54 | 34 | 45 |
| N | .69 | .91 | 1.02 | .90 | .89 | .67 | .82 | 1.00 | .44 | .70 |
|  | (7) Always Uninsured | | | | | | | | | |
| Pct | 19.53 | 22.06 | 20.61 | 22.20 | 19.17 | 16.46 | 19.88 | 13.82 | 10.89 | 9.26 |
| SE | (1.42) | (1.75) | (1.39) | (1.59) | (1.42) | (1.16) | (1.45) | (1.31) | (0.92) | (1.03) |
| n | 317 | 250 | 417 | 370 | 332 | 405 | 438 | 290 | 204 | 191 |
| N | 5.58 | 5.88 | 5.94 | 6.47 | 5.54 | 5.01 | 5.84 | 3.99 | 3.26 | 2.82 |
|  | (8) Any Insurance Interruption, i.e. Patterns Two to Seven | | | | | | | | | |
| Pct | 55.57 | 59.85 | 59.37 | 53.82 | 49.64 | 48.41 | 54.11 | 46.58 | 41.60 | 36.85 |
| SE | (1.69) | (2.11) | (1.84) | (1.94) | (2.22) | (1.80) | (1.95) | (2.00) | (1.99) | (1.85) |
| n | 802 | 632 | 1090 | 850 | 736 | 1022 | 1028 | 851 | 686 | 665 |
| N | 15.86 | 15.94 | 17.10 | 15.69 | 14.34 | 14.72 | 15.89 | 13.45 | 12.48 | 11.22 |
| ^a^Percentage of a pattern in the total seven patterns; ^b^Standard Error of the percentage;  ^c^Number of observations in the sample; ^d^Sample-weighted population in millions for a pattern. | | | | | | | | | | |

| **Table A4 Distributions of Insurance Patterns from 2006 to 2016, Age 26 to 64** | | | | | | | | | | |
| --- | --- | --- | --- | --- | --- | --- | --- | --- | --- | --- |
| Year | 2006-2007 | 2007-2008 | 2008-2009 | 2009-2010 | 2010-2011 | 2011-2012 | 2012-2013 | 2013-2014 | 2014-2015 | 2015-2016 |
|  | (1) Always Insured | | | | | | | | | |
| Pct^a^ | 69.91 | 67.39 | 66.72 | 68.35 | 68.02 | 66.92 | 65.79 | 66.80 | 69.98 | 73.42 |
| SE^b^ | (0.76) | (0.94) | (0.92) | (0.87) | (0.83) | (0.88) | (0.99) | (0.97) | (0.88) | (0.83) |
| n^c^ | 4972 | 3771 | 5152 | 4878 | 4186 | 5220 | 4946 | 4621 | 4750 | 5350 |
| N^d^ | 106.15 | 104.63 | 103.12 | 106.00 | 106.71 | 104.62 | 103.90 | 106.97 | 111.93 | 117.19 |
|  | (2) Single Gap | | | | | | | | | |
| Pct | 3.47 | 4.20 | 4.06 | 3.78 | 4.20 | 3.58 | 3.56 | 5.16 | 4.53 | 4.77 |
| SE | (0.25) | (0.31) | (0.35) | (0.31) | (0.34) | (0.29) | (0.33) | (0.37) | (0.42) | (0.40) |
| n | 282 | 238 | 319 | 287 | 265 | 316 | 308 | 386 | 348 | 387 |
| N | 5.27 | 6.52 | 6.27 | 5.86 | 6.60 | 5.60 | 5.63 | 8.26 | 7.24 | 7.62 |
|  | (3) Transition Into Coverage | | | | | | | | | |
| Pct | 6.91 | 7.67 | 6.22 | 5.71 | 6.94 | 6.65 | 7.59 | 10.03 | 9.88 | 7.03 |
| SE | (0.40) | (0.44) | (0.36) | (0.35) | (0.41) | (0.36) | (0.45) | (0.51) | (0.54) | (0.37) |
| n | 583 | 472 | 593 | 491 | 512 | 654 | 721 | 930 | 911 | 702 |
| N | 10.50 | 11.91 | 9.61 | 8.85 | 10.89 | 10.39 | 11.99 | 16.05 | 15.80 | 11.22 |
|  | (4) Transition Out of Coverage | | | | | | | | | |
| Pct | 4.95 | 4.86 | 5.59 | 5.37 | 4.76 | 5.06 | 5.14 | 3.53 | 3.29 | 3.37 |
| SE | (0.35) | (0.33) | (0.36) | (0.38) | (0.34) | (0.36) | (0.35) | (0.27) | (0.32) | (0.26) |
| n | 421 | 331 | 543 | 467 | 376 | 466 | 483 | 330 | 274 | 326 |
| N | 7.52 | 7.54 | 8.65 | 8.32 | 7.47 | 7.90 | 8.12 | 5.65 | 5.26 | 5.38 |
|  | (5) Temporary Coverage | | | | | | | | | |
| Pct | 0.97 | 1.33 | 1.30 | 1.14 | 1.07 | 1.41 | 1.18 | 0.95 | 1.36 | 1.46 |
| SE | (0.15) | (0.17) | (0.16) | (0.13) | (0.12) | (0.18) | (0.14) | (0.12) | (0.16) | (0.16) |
| n | 100 | 96 | 147 | 119 | 107 | 146 | 128 | 104 | 145 | 162 |
| N | 1.47 | 2.06 | 2.01 | 1.77 | 1.68 | 2.21 | 1.86 | 1.52 | 2.17 | 2.34 |
|  | (6) Repeatedly Uninsured | | | | | | | | | |
| Pct | 1.00 | 1.47 | 1.41 | 1.14 | 1.14 | 1.24 | 1.07 | 1.34 | 1.83 | 1.56 |
| SE | (0.14) | (0.17) | (0.18) | (0.16) | (0.14) | (0.15) | (0.16) | (0.17) | (0.20) | (0.17) |
| n | 101 | 82 | 125 | 96 | 99 | 112 | 118 | 139 | 152 | 140 |
| N | 1.52 | 2.29 | 2.19 | 1.77 | 1.78 | 1.93 | 1.70 | 2.15 | 2.93 | 2.48 |
|  | (7) Always Uninsured | | | | | | | | | |
| Pct | 12.78 | 13.08 | 14.69 | 14.51 | 13.87 | 15.14 | 15.66 | 12.20 | 9.13 | 8.38 |
| SE | (0.53) | (0.67) | (0.63) | (0.67) | (0.57) | (0.64) | (0.75) | (0.64) | (0.55) | (0.46) |
| n | 1317 | 952 | 1714 | 1458 | 1374 | 1834 | 1908 | 1423 | 1078 | 1066 |
| N | 19.40 | 20.31 | 22.71 | 22.50 | 21.75 | 23.68 | 24.73 | 19.53 | 14.60 | 13.38 |
|  | (8) Any Insurance Interruption, i.e. Patterns Two to Seven | | | | | | | | | |
| Pct | 30.09 | 32.61 | 33.28 | 31.65 | 31.98 | 33.08 | 34.21 | 33.20 | 30.02 | 26.58 |
| SE | (0.76) | (0.94) | (0.92) | (0.87) | (0.83) | (0.88) | (0.99) | (0.97) | (0.88) | (0.83) |
| n | 2804 | 2171 | 3441 | 2918 | 2733 | 3528 | 3666 | 3312 | 2908 | 2783 |
| N | 45.68 | 50.63 | 51.43 | 49.07 | 50.17 | 51.72 | 54.03 | 53.15 | 48.01 | 42.42 |
| ^a^Percentage of a pattern in the total seven patterns; ^b^Standard Error of the percentage;  ^c^Number of observations in the sample; ^d^Sample-weighted population in millions for a pattern. | | | | | | | | | | |

| **Table A5 Distributions of Insurance Patterns from 2006 to 2016, Low Income (<200% FPL)** | | | | | | | | | | |
| --- | --- | --- | --- | --- | --- | --- | --- | --- | --- | --- |
| Year | 2006-2007 | 2007-2008 | 2008-2009 | 2009-2010 | 2010-2011 | 2011-2012 | 2012-2013 | 2013-2014 | 2014-2015 | 2015-2016 |
|  | (1) Always Insured | | | | | | | | | |
| Pct^a^ | 50.63 | 47.32 | 47.59 | 52.20 | 52.10 | 54.22 | 51.29 | 53.07 | 56.78 | 60.93 |
| SE^b^ | (1.20) | (1.62) | (1.38) | (1.37) | (1.24) | (1.14) | (1.13) | (1.14) | (1.39) | (1.29) |
| n^c^ | 3042 | 2078 | 3473 | 3126 | 2865 | 4057 | 3934 | 3812 | 3594 | 3928 |
| N^d^ | 40.76 | 37.71 | 39.92 | 46.46 | 47.59 | 51.29 | 48.58 | 48.78 | 50.78 | 50.08 |
|  | (2) Single Gap | | | | | | | | | |
| Pct | 6.07 | 5.73 | 6.78 | 5.77 | 6.39 | 5.58 | 4.95 | 6.95 | 6.13 | 7.09 |
| SE | (0.53) | (0.64) | (0.60) | (0.50) | (0.58) | (0.45) | (0.38) | (0.64) | (0.54) | (0.63) |
| n | 374 | 247 | 451 | 353 | 327 | 414 | 387 | 472 | 377 | 381 |
| N | 4.88 | 4.57 | 5.69 | 5.14 | 5.84 | 5.28 | 4.69 | 6.39 | 5.48 | 5.83 |
|  | (3) Transition Into Coverage | | | | | | | | | |
| Pct | 9.66 | 12.44 | 10.79 | 8.78 | 9.64 | 9.49 | 11.88 | 12.59 | 13.91 | 9.82 |
| SE | (0.63) | (0.99) | (0.66) | (0.58) | (0.65) | (0.55) | (0.75) | (0.60) | (0.81) | (0.68) |
| n | 572 | 495 | 752 | 540 | 551 | 697 | 801 | 844 | 863 | 592 |
| N | 7.77 | 9.92 | 9.05 | 7.82 | 8.81 | 8.98 | 11.25 | 11.57 | 12.44 | 8.07 |
|  | (4) Transition Out of Coverage | | | | | | | | | |
| Pct | 8.52 | 8.44 | 7.48 | 6.71 | 7.00 | 6.52 | 6.86 | 5.17 | 5.04 | 5.62 |
| SE | (0.60) | (0.63) | (0.52) | (0.45) | (0.46) | (0.47) | (0.49) | (0.39) | (0.53) | (0.48) |
| n | 500 | 349 | 511 | 426 | 401 | 497 | 519 | 364 | 320 | 335 |
| N | 6.86 | 6.72 | 6.28 | 5.97 | 6.40 | 6.16 | 6.50 | 4.75 | 4.50 | 4.62 |
|  | (5) Temporary Coverage | | | | | | | | | |
| Pct | 2.30 | 2.75 | 2.73 | 1.96 | 2.04 | 2.28 | 2.05 | 1.97 | 2.52 | 2.65 |
| SE | (0.28) | (0.32) | (0.35) | (0.23) | (0.23) | (0.24) | (0.36) | (0.22) | (0.35) | (0.30) |
| n | 152 | 114 | 192 | 131 | 119 | 173 | 136 | 143 | 143 | 159 |
| N | 1.85 | 2.19 | 2.29 | 1.75 | 1.86 | 2.16 | 1.94 | 1.81 | 2.25 | 2.18 |
|  | (6) Repeatedly Uninsured | | | | | | | | | |
| Pct | 2.81 | 2.81 | 3.34 | 2.74 | 2.64 | 2.00 | 2.34 | 2.95 | 2.44 | 2.69 |
| SE | (0.39) | (0.35) | (0.37) | (0.39) | (0.36) | (0.23) | (0.29) | (0.36) | (0.24) | (0.37) |
| n | 190 | 121 | 200 | 164 | 143 | 152 | 175 | 175 | 172 | 154 |
| N | 2.26 | 2.24 | 2.80 | 2.44 | 2.41 | 1.89 | 2.22 | 2.71 | 2.18 | 2.21 |
|  | (7) Always Uninsured | | | | | | | | | |
| Pct | 20.01 | 20.51 | 21.29 | 21.83 | 20.19 | 19.91 | 20.64 | 17.32 | 13.17 | 11.18 |
| SE | (0.87) | (1.10) | (1.05) | (1.16) | (0.99) | (0.89) | (0.88) | (0.95) | (0.85) | (0.75) |
| n | 1256 | 890 | 1542 | 1332 | 1251 | 1619 | 1673 | 1274 | 938 | 895 |
| N | 16.11 | 16.35 | 17.86 | 19.43 | 18.44 | 18.83 | 19.55 | 15.92 | 11.78 | 9.19 |
|  | (8) Any Insurance Interruption, i.e. Patterns Two to Seven | | | | | | | | | |
| Pct | 49.37 | 52.68 | 52.41 | 47.80 | 47.90 | 45.78 | 48.71 | 46.93 | 43.22 | 39.07 |
| SE | (1.20) | (1.62) | (1.38) | (1.37) | (1.24) | (1.14) | (1.13) | (1.14) | (1.39) | (1.29) |
| n | 3044 | 2216 | 3648 | 2946 | 2792 | 3552 | 3691 | 3272 | 2813 | 2516 |
| N | 39.74 | 41.99 | 43.97 | 42.54 | 43.76 | 43.30 | 46.14 | 43.14 | 38.65 | 32.11 |
| ^a^Percentage of a pattern in the total seven patterns; ^b^Standard Error of the percentage;  ^c^Number of observations in the sample; ^d^Sample-weighted population in millions for a pattern. | | | | | | | | | | |

| **Table A6 Distributions of Insurance Patterns from 2006 to 2016, Middle Income (200% to 399% FPL)** | | | | | | | | | | |
| --- | --- | --- | --- | --- | --- | --- | --- | --- | --- | --- |
| Year | 2006-2007 | 2007-2008 | 2008-2009 | 2009-2010 | 2010-2011 | 2011-2012 | 2012-2013 | 2013-2014 | 2014-2015 | 2015-2016 |
|  | (1) Always Insured | | | | | | | | | |
| Pct^a^ | 67.32 | 65.68 | 64.65 | 68.31 | 69.77 | 68.28 | 66.60 | 69.14 | 69.67 | 72.78 |
| SE^b^ | (1.40) | (1.32) | (1.26) | (1.40) | (1.36) | (1.64) | (1.41) | (1.44) | (1.30) | (1.39) |
| n^c^ | 2451 | 2090 | 2882 | 2747 | 2385 | 2838 | 2551 | 2446 | 2520 | 2783 |
| N^d^ | 52.59 | 56.83 | 53.75 | 54.80 | 56.78 | 54.54 | 53.11 | 52.54 | 55.41 | 58.29 |
|  | (2) Single Gap | | | | | | | | | |
| Pct | 4.78 | 5.96 | 5.64 | 5.02 | 4.89 | 4.91 | 5.22 | 5.92 | 6.40 | 5.27 |
| SE | (0.51) | (0.75) | (0.66) | (0.67) | (0.56) | (0.63) | (0.68) | (0.63) | (0.87) | (0.65) |
| n | 196 | 187 | 229 | 198 | 164 | 204 | 201 | 206 | 220 | 224 |
| N | 3.73 | 5.15 | 4.69 | 4.03 | 3.98 | 3.92 | 4.16 | 4.50 | 5.09 | 4.22 |
|  | (3) Transition Into Coverage | | | | | | | | | |
| Pct | 7.61 | 9.00 | 6.92 | 5.92 | 7.29 | 6.33 | 7.16 | 10.07 | 9.57 | 7.20 |
| SE | (0.66) | (0.82) | (0.56) | (0.65) | (0.64) | (0.63) | (0.60) | (0.78) | (0.68) | (0.56) |
| n | 311 | 282 | 340 | 251 | 260 | 317 | 319 | 423 | 409 | 360 |
| N | 5.94 | 7.78 | 5.76 | 4.75 | 5.93 | 5.06 | 5.71 | 7.65 | 7.61 | 5.76 |
|  | (4) Transition Out of Coverage | | | | | | | | | |
| Pct | 6.18 | 5.42 | 7.36 | 6.75 | 5.18 | 6.03 | 6.60 | 3.26 | 4.05 | 4.52 |
| SE | (0.72) | (0.51) | (0.57) | (0.70) | (0.48) | (0.61) | (0.66) | (0.37) | (0.51) | (0.51) |
| n | 248 | 202 | 349 | 280 | 206 | 260 | 280 | 152 | 162 | 205 |
| N | 4.83 | 4.69 | 6.12 | 5.41 | 4.21 | 4.81 | 5.26 | 2.48 | 3.22 | 3.62 |
|  | (5) Temporary Coverage | | | | | | | | | |
| Pct | 1.02 | 1.19 | 1.51 | 1.11 | 0.75 | 1.67 | 1.56 | 0.93 | 1.19 | 1.39 |
| SE | (0.22) | (0.31) | (0.30) | (0.18) | (0.15) | (0.41) | (0.32) | (0.21) | (0.20) | (0.22) |
| n | 43 | 36 | 73 | 62 | 37 | 67 | 65 | 40 | 61 | 69 |
| N | .79 | 1.03 | 1.25 | .89 | .61 | 1.33 | 1.24 | .71 | .95 | 1.11 |
|  | (6) Repeatedly Uninsured | | | | | | | | | |
| Pct | 1.20 | 1.64 | 1.49 | 1.44 | 1.40 | 1.67 | 1.17 | 1.54 | 1.68 | 1.59 |
| SE | (0.24) | (0.30) | (0.25) | (0.27) | (0.26) | (0.31) | (0.22) | (0.28) | (0.33) | (0.33) |
| n | 59 | 51 | 85 | 57 | 51 | 68 | 59 | 77 | 55 | 72 |
| N | .94 | 1.42 | 1.24 | 1.16 | 1.14 | 1.33 | .93 | 1.17 | 1.34 | 1.27 |
|  | (7) Always Uninsured | | | | | | | | | |
| Pct | 11.90 | 11.12 | 12.43 | 11.44 | 10.71 | 11.12 | 11.69 | 9.14 | 7.45 | 7.26 |
| SE | (0.85) | (0.93) | (0.91) | (0.73) | (0.86) | (0.77) | (0.81) | (0.82) | (0.78) | (0.72) |
| n | 511 | 390 | 660 | 548 | 500 | 622 | 646 | 462 | 377 | 358 |
| N | 9.29 | 9.63 | 10.33 | 9.18 | 8.72 | 8.89 | 9.32 | 6.94 | 5.92 | 5.81 |
|  | (8) Any Insurance Interruption, i.e. Patterns Two to Seven | | | | | | | | | |
| Pct | 32.68 | 34.32 | 35.35 | 31.69 | 30.23 | 31.72 | 33.40 | 30.86 | 30.33 | 27.22 |
| SE | (1.40) | (1.32) | (1.26) | (1.40) | (1.36) | (1.64) | (1.41) | (1.44) | (1.30) | (1.39) |
| n | 1368 | 1148 | 1736 | 1396 | 1218 | 1538 | 1570 | 1360 | 1284 | 1288 |
| N | 25.53 | 29.70 | 29.40 | 25.42 | 24.60 | 25.34 | 26.64 | 23.45 | 24.12 | 21.80 |
| ^a^Percentage of a pattern in the total seven patterns; ^b^Standard Error of the percentage;  ^c^Number of observations in the sample; ^d^Sample-weighted population in millions for a pattern. | | | | | | | | | | |

| **Table A7 Distributions of Insurance Patterns from 2006 to 2016, High Income (400% FPL and Above)** | | | | | | | | | | |
| --- | --- | --- | --- | --- | --- | --- | --- | --- | --- | --- |
| Year | 2006-2007 | 2007-2008 | 2008-2009 | 2009-2010 | 2010-2011 | 2011-2012 | 2012-2013 | 2013-2014 | 2014-2015 | 2015-2016 |
|  | (1) Always Insured | | | | | | | | | |
| Pct^a^ | 84.07 | 82.51 | 82.58 | 84.28 | 85.22 | 85.26 | 85.04 | 84.31 | 87.43 | 86.29 |
| SE^b^ | (0.93) | (1.09) | (1.13) | (0.99) | (1.09) | (1.02) | (0.91) | (1.28) | (1.03) | (1.22) |
| n^c^ | 3275 | 2420 | 3147 | 2873 | 2462 | 2997 | 2918 | 2722 | 2719 | 3038 |
| N^d^ | 80.33 | 74.33 | 75.22 | 75.72 | 74.67 | 73.80 | 74.78 | 81.17 | 83.77 | 88.13 |
|  | (2) Single Gap | | | | | | | | | |
| Pct | 3.57 | 3.68 | 3.36 | 2.93 | 2.69 | 2.56 | 2.99 | 4.38 | 3.50 | 3.89 |
| SE | (0.48) | (0.48) | (0.59) | (0.46) | (0.41) | (0.37) | (0.45) | (0.66) | (0.63) | (0.88) |
| n | 149 | 109 | 105 | 102 | 81 | 101 | 111 | 133 | 111 | 130 |
| N | 3.41 | 3.32 | 3.06 | 2.63 | 2.36 | 2.22 | 2.63 | 4.22 | 3.35 | 3.97 |
|  | (3) Transition Into Coverage | | | | | | | | | |
| Pct | 4.35 | 4.45 | 4.18 | 3.33 | 4.34 | 3.82 | 3.16 | 4.65 | 3.04 | 3.09 |
| SE | (0.49) | (0.50) | (0.46) | (0.54) | (0.59) | (0.53) | (0.44) | (0.61) | (0.46) | (0.39) |
| n | 174 | 136 | 196 | 126 | 135 | 152 | 137 | 176 | 119 | 154 |
| N | 4.16 | 4.01 | 3.81 | 2.99 | 3.81 | 3.31 | 2.78 | 4.47 | 2.91 | 3.16 |
|  | (4) Transition Out of Coverage | | | | | | | | | |
| Pct | 4.00 | 3.17 | 3.20 | 4.05 | 2.90 | 3.46 | 3.12 | 2.80 | 2.95 | 2.36 |
| SE | (0.50) | (0.39) | (0.43) | (0.63) | (0.58) | (0.42) | (0.42) | (0.43) | (0.50) | (0.36) |
| n | 156 | 103 | 143 | 134 | 95 | 137 | 128 | 110 | 94 | 99 |
| N | 3.83 | 2.86 | 2.91 | 3.64 | 2.54 | 3.00 | 2.74 | 2.70 | 2.83 | 2.41 |
|  | (5) Temporary Coverage | | | | | | | | | |
| Pct | 0.37 | 0.56 | 0.82 | 0.45 | 0.27 | 0.41 | 0.49 | 0.26 | 0.38 | 0.44 |
| SE | (0.11) | (0.18) | (0.24) | (0.19) | (0.08) | (0.16) | (0.12) | (0.12) | (0.14) | (0.17) |
| n | 17 | 18 | 36 | 20 | 14 | 12 | 26 | 7 | 19 | 22 |
| N | .36 | .51 | .75 | .40 | .24 | .35 | .43 | .25 | .37 | .45 |
|  | (6) Repeatedly Uninsured | | | | | | | | | |
| Pct | 0.42 | 0.78 | 0.69 | 0.53 | 0.45 | 0.37 | 0.31 | 0.55 | 0.60 | 0.84 |
| SE | (0.13) | (0.22) | (0.16) | (0.16) | (0.13) | (0.11) | (0.14) | (0.14) | (0.16) | (0.20) |
| n | 18 | 23 | 30 | 15 | 18 | 19 | 15 | 30 | 23 | 34 |
| N | .40 | .71 | .63 | .47 | .40 | .32 | .27 | .53 | .57 | .85 |
|  | (7) Always Uninsured | | | | | | | | | |
| Pct | 3.21 | 4.84 | 5.17 | 4.43 | 4.12 | 4.12 | 4.90 | 3.05 | 2.10 | 3.09 |
| SE | (0.42) | (0.63) | (0.56) | (0.51) | (0.43) | (0.46) | (0.59) | (0.49) | (0.33) | (0.45) |
| n | 140 | 156 | 233 | 194 | 175 | 187 | 242 | 139 | 94 | 132 |
| N | 3.07 | 4.36 | 4.71 | 3.98 | 3.61 | 3.57 | 4.31 | 2.94 | 2.01 | 3.15 |
|  | (8) Any Insurance Interruption, i.e. Patterns Two to Seven | | | | | | | | | |
| Pct | 15.93 | 17.49 | 17.42 | 15.72 | 14.78 | 14.74 | 14.96 | 15.69 | 12.57 | 13.71 |
| SE | (0.93) | (1.09) | (1.13) | (0.99) | (1.09) | (1.02) | (0.91) | (1.28) | (1.03) | (1.22) |
| n | 654 | 545 | 743 | 591 | 518 | 608 | 659 | 595 | 460 | 571 |
| N | 15.22 | 15.76 | 15.86 | 14.12 | 12.95 | 12.76 | 13.15 | 15.10 | 12.04 | 14.00 |
| ^a^Percentage of a pattern in the total seven patterns; ^b^Standard Error of the percentage;  ^c^Number of observations in the sample; ^d^Sample-weighted population in millions for a pattern. | | | | | | | | | | |

| **Table A8 Distributions of Insurance Patterns from 2006 to 2016, Non-Hispanic White** | | | | | | | | | | |
| --- | --- | --- | --- | --- | --- | --- | --- | --- | --- | --- |
| Year | 2006-2007 | 2007-2008 | 2008-2009 | 2009-2010 | 2010-2011 | 2011-2012 | 2012-2013 | 2013-2014 | 2014-2015 | 2015-2016 |
|  | (1) Always Insured | | | | | | | | | |
| Pct^a^ | 73.52 | 70.62 | 70.82 | 73.43 | 73.87 | 74.43 | 72.82 | 74.42 | 77.61 | 79.25 |
| SE^b^ | (0.88) | (1.06) | (1.09) | (1.06) | (1.05) | (1.03) | (1.07) | (1.10) | (1.04) | (0.97) |
| n^c^ | 4797 | 3573 | 4031 | 4224 | 3424 | 4408 | 3567 | 3455 | 3393 | 3805 |
| N^d^ | 119.38 | 115.76 | 115.50 | 119.65 | 118.11 | 119.50 | 115.74 | 116.22 | 118.61 | 120.57 |
|  | (2) Single Gap | | | | | | | | | |
| Pct | 4.41 | 5.14 | 5.42 | 4.67 | 4.96 | 3.82 | 4.00 | 5.78 | 5.05 | 5.36 |
| SE | (0.36) | (0.46) | (0.50) | (0.43) | (0.43) | (0.38) | (0.50) | (0.61) | (0.61) | (0.67) |
| n | 293 | 268 | 319 | 290 | 252 | 246 | 205 | 309 | 226 | 274 |
| N | 7.17 | 8.42 | 8.84 | 7.61 | 7.92 | 6.14 | 6.37 | 9.03 | 7.72 | 8.16 |
|  | (3) Transition Into Coverage | | | | | | | | | |
| Pct | 5.70 | 7.79 | 5.72 | 5.15 | 6.55 | 5.80 | 6.75 | 7.80 | 7.05 | 4.98 |
| SE | (0.49) | (0.58) | (0.49) | (0.47) | (0.46) | (0.45) | (0.56) | (0.51) | (0.56) | (0.40) |
| n | 371 | 374 | 358 | 292 | 338 | 369 | 343 | 403 | 370 | 295 |
| N | 9.26 | 12.78 | 9.34 | 8.39 | 10.48 | 9.32 | 10.73 | 12.18 | 10.78 | 7.57 |
|  | (4) Transition Out of Coverage | | | | | | | | | |
| Pct | 5.74 | 5.22 | 5.64 | 5.31 | 4.60 | 4.99 | 5.08 | 2.99 | 3.62 | 3.36 |
| SE | (0.47) | (0.37) | (0.42) | (0.47) | (0.43) | (0.39) | (0.46) | (0.31) | (0.45) | (0.37) |
| n | 392 | 288 | 326 | 311 | 249 | 311 | 273 | 165 | 163 | 172 |
| N | 9.31 | 8.55 | 9.20 | 8.66 | 7.35 | 8.01 | 8.08 | 4.67 | 5.54 | 5.11 |
|  | (5) Temporary Coverage | | | | | | | | | |
| Pct | 0.86 | 1.15 | 1.52 | 0.91 | 0.65 | 1.34 | 1.22 | 0.72 | 1.02 | 1.00 |
| SE | (0.15) | (0.18) | (0.24) | (0.16) | (0.11) | (0.23) | (0.26) | (0.13) | (0.18) | (0.16) |
| n | 58 | 59 | 89 | 63 | 50 | 85 | 65 | 41 | 59 | 63 |
| N | 1.39 | 1.88 | 2.48 | 1.48 | 1.04 | 2.16 | 1.94 | 1.13 | 1.56 | 1.52 |
|  | (6) Repeatedly Uninsured | | | | | | | | | |
| Pct | 1.27 | 1.58 | 1.51 | 1.38 | 1.32 | 1.17 | 1.06 | 1.52 | 1.25 | 1.29 |
| SE | (0.21) | (0.20) | (0.22) | (0.20) | (0.23) | (0.16) | (0.17) | (0.23) | (0.22) | (0.22) |
| n | 95 | 79 | 88 | 86 | 69 | 82 | 66 | 92 | 58 | 73 |
| N | 2.07 | 2.59 | 2.47 | 2.25 | 2.10 | 1.88 | 1.68 | 2.38 | 1.90 | 1.96 |
|  | (7) Always Uninsured | | | | | | | | | |
| Pct | 8.50 | 8.50 | 9.36 | 9.16 | 8.06 | 8.43 | 9.06 | 6.76 | 4.39 | 4.76 |
| SE | (0.53) | (0.66) | (0.64) | (0.58) | (0.50) | (0.52) | (0.65) | (0.62) | (0.49) | (0.45) |
| n | 564 | 456 | 569 | 564 | 503 | 580 | 489 | 372 | 221 | 269 |
| N | 13.80 | 13.94 | 15.26 | 14.92 | 12.89 | 13.54 | 14.40 | 10.56 | 6.72 | 7.24 |
|  | (8) Any Insurance Interruption, i.e. Patterns Two to Seven | | | | | | | | | |
| Pct | 26.48 | 29.38 | 29.18 | 26.57 | 26.13 | 25.57 | 27.18 | 25.58 | 22.39 | 20.75 |
| SE | (0.88) | (1.06) | (1.09) | (1.06) | (1.05) | (1.03) | (1.07) | (1.10) | (1.04) | (0.97) |
| n | 1773 | 1524 | 1749 | 1606 | 1461 | 1673 | 1441 | 1382 | 1097 | 1146 |
| N | 42.99 | 48.15 | 47.58 | 43.31 | 41.79 | 41.05 | 43.19 | 39.95 | 34.21 | 31.57 |
| ^a^Percentage of a pattern in the total seven patterns; ^b^Standard Error of the percentage;  ^c^Number of observations in the sample; ^d^Sample-weighted population in millions for a pattern. | | | | | | | | | | |

| **Table A9 Distributions of Insurance Patterns from 2006 to 2016, Non-Hispanic Black** | | | | | | | | | | |
| --- | --- | --- | --- | --- | --- | --- | --- | --- | --- | --- |
| Year | 2006-2007 | 2007-2008 | 2008-2009 | 2009-2010 | 2010-2011 | 2011-2012 | 2012-2013 | 2013-2014 | 2014-2015 | 2015-2016 |
|  | (1) Always Insured | | | | | | | | | |
| Pct^a^ | 65.56 | 63.74 | 61.86 | 65.82 | 65.03 | 62.87 | 60.94 | 65.16 | 67.51 | 70.15 |
| SE^b^ | (1.50) | (1.82) | (1.24) | (1.46) | (1.48) | (1.31) | (1.51) | (1.30) | (1.21) | (1.42) |
| n^c^ | 1485 | 1129 | 2071 | 1728 | 1615 | 1983 | 1948 | 1946 | 1817 | 1862 |
| N^d^ | 21.15 | 20.72 | 19.89 | 21.47 | 21.16 | 20.66 | 19.95 | 21.80 | 22.87 | 23.72 |
|  | (2) Single Gap | | | | | | | | | |
| Pct | 4.85 | 5.39 | 4.58 | 3.62 | 5.08 | 5.69 | 6.76 | 5.62 | 5.91 | 5.58 |
| SE | (0.63) | (0.75) | (0.55) | (0.47) | (0.67) | (0.62) | (0.81) | (0.63) | (0.90) | (0.71) |
| n | 130 | 97 | 154 | 106 | 128 | 175 | 195 | 177 | 143 | 143 |
| N | 1.57 | 1.75 | 1.47 | 1.18 | 1.65 | 1.87 | 2.21 | 1.88 | 2.00 | 1.89 |
|  | (3) Transition Into Coverage | | | | | | | | | |
| Pct | 9.23 | 9.10 | 9.14 | 7.64 | 8.99 | 8.84 | 9.28 | 10.27 | 10.30 | 8.72 |
| SE | (0.90) | (1.08) | (0.69) | (0.65) | (0.86) | (0.74) | (0.70) | (0.66) | (0.67) | (0.81) |
| n | 208 | 159 | 297 | 227 | 224 | 298 | 302 | 326 | 306 | 220 |
| N | 2.98 | 2.96 | 2.94 | 2.49 | 2.93 | 2.90 | 3.04 | 3.44 | 3.49 | 2.95 |
|  | (4) Transition Out of Coverage | | | | | | | | | |
| Pct | 6.70 | 5.63 | 7.68 | 7.68 | 5.97 | 6.25 | 6.27 | 5.14 | 4.43 | 5.14 |
| SE | (0.78) | (0.69) | (0.65) | (0.75) | (0.73) | (0.65) | (0.48) | (0.66) | (0.64) | (0.62) |
| n | 170 | 120 | 256 | 201 | 162 | 204 | 211 | 160 | 131 | 148 |
| N | 2.16 | 1.83 | 2.47 | 2.50 | 1.94 | 2.05 | 2.05 | 1.72 | 1.50 | 1.74 |
|  | (5) Temporary Coverage | | | | | | | | | |
| Pct | 1.72 | 1.67 | 1.43 | 1.43 | 1.79 | 1.54 | 1.69 | 1.51 | 1.51 | 1.59 |
| SE | (0.36) | (0.42) | (0.22) | (0.28) | (0.38) | (0.27) | (0.28) | (0.30) | (0.31) | (0.24) |
| n | 44 | 29 | 51 | 40 | 42 | 51 | 46 | 49 | 44 | 46 |
| N | .55 | .54 | .46 | .47 | .58 | .50 | .55 | .51 | .51 | .54 |
|  | (6) Repeatedly Uninsured | | | | | | | | | |
| Pct | 1.15 | 1.94 | 2.02 | 1.52 | 2.10 | 1.83 | 2.26 | 2.43 | 2.03 | 2.23 |
| SE | (0.25) | (0.49) | (0.36) | (0.26) | (0.34) | (0.34) | (0.51) | (0.44) | (0.29) | (0.43) |
| n | 35 | 35 | 64 | 44 | 49 | 57 | 69 | 70 | 58 | 60 |
| N | .37 | .63 | .65 | .49 | .68 | .60 | .74 | .81 | .69 | .75 |
|  | (7) Always Uninsured | | | | | | | | | |
| Pct | 10.80 | 12.54 | 13.29 | 12.29 | 11.04 | 12.99 | 12.81 | 9.87 | 8.32 | 6.59 |
| SE | (0.85) | (1.24) | (0.93) | (0.83) | (0.80) | (0.90) | (0.82) | (0.65) | (0.82) | (0.70) |
| n | 256 | 233 | 405 | 325 | 288 | 415 | 417 | 331 | 252 | 184 |
| N | 3.48 | 4.08 | 4.27 | 4.01 | 3.59 | 4.27 | 4.19 | 3.30 | 2.82 | 2.23 |
|  | (8) Any Insurance Interruption, i.e. Patterns Two to Seven | | | | | | | | | |
| Pct | 34.44 | 36.26 | 38.14 | 34.18 | 34.97 | 37.13 | 39.06 | 34.84 | 32.49 | 29.85 |
| SE | (1.50) | (1.82) | (1.24) | (1.46) | (1.48) | (1.31) | (1.51) | (1.30) | (1.21) | (1.42) |
| n | 843 | 673 | 1227 | 943 | 893 | 1200 | 1240 | 1113 | 934 | 801 |
| N | 11.11 | 11.79 | 12.26 | 11.15 | 11.38 | 12.20 | 12.79 | 11.66 | 11.00 | 10.09 |
| ^a^Percentage of a pattern in the total seven patterns; ^b^Standard Error of the percentage;  ^c^Number of observations in the sample; ^d^Sample-weighted population in millions for a pattern. | | | | | | | | | | |

| **Table A10 Distributions of Insurance Patterns from 2006 to 2016, Hispanic** | | | | | | | | | | |
| --- | --- | --- | --- | --- | --- | --- | --- | --- | --- | --- |
| Year | 2006-2007 | 2007-2008 | 2008-2009 | 2009-2010 | 2010-2011 | 2011-2012 | 2012-2013 | 2013-2014 | 2014-2015 | 2015-2016 |
|  | (1) Always Insured | | | | | | | | | |
| Pct^a^ | 49.06 | 46.90 | 47.46 | 51.06 | 52.27 | 54.65 | 51.41 | 52.62 | 55.10 | 60.96 |
| SE^b^ | (1.62) | (2.04) | (1.56) | (1.81) | (1.41) | (1.42) | (1.37) | (1.66) | (1.31) | (1.58) |
| n^c^ | 1832 | 1255 | 2389 | 1905 | 1771 | 2605 | 2753 | 2476 | 2491 | 2900 |
| N^d^ | 20.10 | 19.79 | 20.67 | 22.86 | 24.57 | 26.18 | 24.98 | 26.37 | 28.02 | 31.55 |
|  | (2) Single Gap | | | | | | | | | |
| Pct | 5.23 | 4.49 | 4.76 | 5.01 | 4.45 | 4.62 | 4.27 | 5.66 | 6.00 | 5.21 |
| SE | (0.55) | (0.73) | (0.46) | (0.56) | (0.54) | (0.47) | (0.36) | (0.66) | (0.67) | (0.61) |
| n | 243 | 132 | 253 | 199 | 156 | 226 | 238 | 237 | 269 | 245 |
| N | 2.14 | 1.89 | 2.07 | 2.24 | 2.09 | 2.21 | 2.08 | 2.84 | 3.05 | 2.70 |
|  | (3) Transition Into Coverage | | | | | | | | | |
| Pct | 10.16 | 11.50 | 10.76 | 7.51 | 8.75 | 7.62 | 8.94 | 11.87 | 12.15 | 9.59 |
| SE | (0.84) | (1.23) | (0.74) | (0.65) | (0.70) | (0.52) | (0.64) | (0.95) | (0.84) | (0.64) |
| n | 411 | 318 | 527 | 312 | 317 | 394 | 486 | 575 | 581 | 493 |
| N | 4.16 | 4.85 | 4.69 | 3.36 | 4.11 | 3.65 | 4.34 | 5.95 | 6.18 | 4.96 |
|  | (4) Transition Out of Coverage | | | | | | | | | |
| Pct | 7.26 | 7.36 | 6.41 | 6.50 | 5.89 | 5.64 | 6.70 | 5.22 | 5.31 | 4.82 |
| SE | (0.68) | (0.77) | (0.50) | (0.60) | (0.55) | (0.47) | (0.47) | (0.55) | (0.58) | (0.47) |
| n | 287 | 213 | 343 | 256 | 224 | 294 | 364 | 238 | 236 | 240 |
| N | 2.97 | 3.11 | 2.79 | 2.91 | 2.77 | 2.70 | 3.25 | 2.62 | 2.70 | 2.49 |
|  | (5) Temporary Coverage | | | | | | | | | |
| Pct | 2.26 | 2.45 | 2.47 | 2.23 | 1.92 | 1.94 | 1.87 | 2.01 | 2.10 | 2.46 |
| SE | (0.35) | (0.37) | (0.32) | (0.29) | (0.29) | (0.31) | (0.28) | (0.30) | (0.34) | (0.40) |
| n | 104 | 69 | 141 | 99 | 67 | 95 | 102 | 93 | 100 | 114 |
| N | .93 | 1.03 | 1.08 | 1.00 | .90 | .93 | .91 | 1.01 | 1.07 | 1.27 |
|  | (6) Repeatedly Uninsured | | | | | | | | | |
| Pct | 2.36 | 2.17 | 2.86 | 2.08 | 2.02 | 1.56 | 1.71 | 1.98 | 2.35 | 2.45 |
| SE | (0.34) | (0.44) | (0.31) | (0.38) | (0.33) | (0.25) | (0.25) | (0.28) | (0.25) | (0.39) |
| n | 127 | 68 | 141 | 72 | 80 | 84 | 97 | 99 | 114 | 111 |
| N | .97 | .92 | 1.25 | .93 | .95 | .75 | .83 | .99 | 1.19 | 1.27 |
|  | (7) Always Uninsured | | | | | | | | | |
| Pct | 23.67 | 25.13 | 25.28 | 25.61 | 24.69 | 23.97 | 25.09 | 20.64 | 17.00 | 14.52 |
| SE | (1.13) | (2.08) | (1.39) | (1.78) | (1.29) | (1.33) | (1.16) | (1.28) | (1.05) | (0.90) |
| n | 1007 | 660 | 1303 | 1042 | 976 | 1290 | 1447 | 1068 | 856 | 871 |
| N | 9.70 | 10.60 | 11.01 | 11.47 | 11.61 | 11.48 | 12.19 | 10.34 | 8.64 | 7.51 |
|  | (8) Any Insurance Interruption, i.e. Patterns Two to Seven | | | | | | | | | |
| Pct | 50.94 | 53.10 | 52.54 | 48.94 | 47.73 | 45.35 | 48.59 | 47.38 | 44.90 | 39.04 |
| SE | (1.62) | (2.04) | (1.56) | (1.81) | (1.41) | (1.42) | (1.37) | (1.66) | (1.31) | (1.58) |
| n | 2179 | 1460 | 2708 | 1980 | 1820 | 2383 | 2734 | 2310 | 2156 | 2074 |
| N | 20.87 | 22.41 | 22.89 | 21.91 | 22.43 | 21.72 | 23.61 | 23.75 | 22.84 | 20.21 |
| ^a^Percentage of a pattern in the total seven patterns; ^b^Standard Error of the percentage;  ^c^Number of observations in the sample; ^d^Sample-weighted population in millions for a pattern. | | | | | | | | | | |

| **Table A11 Distributions of Insurance Patterns from 2006 to 2016, Non-Hispanic Other Races** | | | | | | | | | | |
| --- | --- | --- | --- | --- | --- | --- | --- | --- | --- | --- |
| Year | 2006-2007 | 2007-2008 | 2008-2009 | 2009-2010 | 2010-2011 | 2011-2012 | 2012-2013 | 2013-2014 | 2014-2015 | 2015-2016 |
|  | (1) Always Insured | | | | | | | | | |
| Pct^a^ | 70.31 | 71.17 | 66.39 | 69.43 | 72.71 | 67.38 | 71.34 | 74.05 | 75.17 | 77.37 |
| SE^b^ | (2.51) | (2.65) | (2.16) | (2.26) | (2.05) | (1.93) | (1.94) | (2.23) | (1.84) | (2.15) |
| n^c^ | 654 | 631 | 1011 | 889 | 902 | 896 | 1135 | 1103 | 1132 | 1182 |
| N^d^ | 13.05 | 12.61 | 12.82 | 12.99 | 15.20 | 13.29 | 15.79 | 18.09 | 20.46 | 20.66 |
|  | (2) Single Gap | | | | | | | | | |
| Pct | 6.21 | 5.52 | 5.46 | 4.08 | 2.43 | 6.07 | 3.74 | 5.51 | 4.23 | 4.80 |
| SE | (1.23) | (1.23) | (1.17) | (0.84) | (0.69) | (0.81) | (0.62) | (1.00) | (0.62) | (0.93) |
| n | 53 | 46 | 59 | 58 | 36 | 72 | 61 | 88 | 70 | 73 |
| N | 1.15 | .98 | 1.05 | .76 | .51 | 1.20 | .83 | 1.35 | 1.15 | 1.28 |
|  | (3) Transition Into Coverage | | | | | | | | | |
| Pct | 7.96 | 6.34 | 8.56 | 7.06 | 4.95 | 7.45 | 7.36 | 8.74 | 9.25 | 5.66 |
| SE | (1.36) | (1.38) | (1.37) | (1.35) | (1.00) | (1.18) | (1.06) | (1.11) | (1.41) | (0.72) |
| n | 67 | 62 | 106 | 86 | 67 | 105 | 126 | 139 | 134 | 98 |
| N | 1.48 | 1.12 | 1.65 | 1.32 | 1.03 | 1.47 | 1.63 | 2.13 | 2.52 | 1.51 |
|  | (4) Transition Out of Coverage | | | | | | | | | |
| Pct | 5.76 | 4.39 | 4.38 | 5.12 | 5.19 | 6.13 | 5.05 | 3.78 | 2.98 | 4.86 |
| SE | (1.07) | (0.93) | (0.85) | (0.92) | (1.13) | (0.88) | (0.87) | (0.79) | (0.68) | (0.69) |
| n | 55 | 33 | 78 | 72 | 67 | 85 | 79 | 63 | 46 | 79 |
| N | 1.07 | .78 | .85 | .96 | 1.08 | 1.21 | 1.12 | .92 | .81 | 1.30 |
|  | (5) Temporary Coverage | | | | | | | | | |
| Pct | 0.71 | 1.55 | 1.44 | 0.49 | 0.91 | 1.31 | 0.97 | 0.50 | 1.59 | 1.52 |
| SE | (0.30) | (0.64) | (0.38) | (0.22) | (0.34) | (0.51) | (0.30) | (0.25) | (0.57) | (0.46) |
| n | 6 | 11 | 20 | 11 | 11 | 21 | 14 | 7 | 20 | 27 |
| N | .13 | .27 | .28 | .09 | .19 | .26 | .21 | .12 | .43 | .41 |
|  | (6) Repeatedly Uninsured | | | | | | | | | |
| Pct | 1.02 | 1.30 | 1.60 | 2.09 | 1.01 | 1.58 | 0.76 | 0.92 | 1.13 | 1.37 |
| SE | (0.33) | (0.46) | (0.42) | (0.54) | (0.33) | (0.70) | (0.27) | (0.33) | (0.29) | (0.53) |
| n | 10 | 13 | 22 | 34 | 14 | 16 | 17 | 21 | 20 | 16 |
| N | .19 | .23 | .31 | .39 | .21 | .31 | .17 | .22 | .31 | .37 |
|  | (7) Always Uninsured | | | | | | | | | |
| Pct | 8.03 | 9.72 | 12.16 | 11.73 | 12.80 | 10.09 | 10.78 | 6.50 | 5.66 | 4.42 |
| SE | (1.25) | (1.73) | (1.70) | (1.49) | (1.59) | (1.34) | (1.22) | (1.07) | (1.15) | (1.16) |
| n | 80 | 87 | 158 | 143 | 159 | 143 | 208 | 104 | 80 | 61 |
| N | 1.49 | 1.72 | 2.35 | 2.20 | 2.68 | 1.99 | 2.39 | 1.59 | 1.54 | 1.18 |
|  | (8) Any Insurance Interruption, i.e. Patterns Two to Seven | | | | | | | | | |
| Pct | 29.69 | 28.83 | 33.61 | 30.57 | 27.29 | 32.62 | 28.66 | 25.95 | 24.83 | 22.63 |
| SE | (2.51) | (2.65) | (2.16) | (2.26) | (2.05) | (1.93) | (1.94) | (2.23) | (1.84) | (2.15) |
| n | 271 | 252 | 443 | 404 | 354 | 442 | 505 | 422 | 370 | 354 |
| N | 5.51 | 5.11 | 6.49 | 5.72 | 5.71 | 6.44 | 6.35 | 6.34 | 6.76 | 6.05 |
| ^a^Percentage of a pattern in the total seven patterns; ^b^Standard Error of the percentage;  ^c^Number of observations in the sample; ^d^Sample-weighted population in millions for a pattern. | | | | | | | | | | |

| **Table A12 Distributions of Insurance Patterns from 2006 to 2016, Northeastern Region** | | | | | | | | | | |
| --- | --- | --- | --- | --- | --- | --- | --- | --- | --- | --- |
| Year | 2006-2007 | 2007-2008 | 2008-2009 | 2009-2010 | 2010-2011 | 2011-2012 | 2012-2013 | 2013-2014 | 2014-2015 | 2015-2016 |
|  | (1) Always Insured | | | | | | | | | |
| Pct^a^ | 74.08 | 69.31 | 74.66 | 73.46 | 74.39 | 74.73 | 73.54 | 76.41 | 78.37 | 81.50 |
| SE^b^ | (1.36) | (1.77) | (1.92) | (2.12) | (2.23) | (2.11) | (1.67) | (2.20) | (1.69) | (1.59) |
| n^c^ | 1375 | 1129 | 1626 | 1377 | 1348 | 1707 | 1720 | 1662 | 1413 | 1643 |
| N^d^ | 34.14 | 31.87 | 34.73 | 33.82 | 34.13 | 34.62 | 34.19 | 35.32 | 36.43 | 37.04 |
|  | (2) Single Gap | | | | | | | | | |
| Pct | 4.30 | 6.40 | 4.97 | 3.68 | 3.33 | 4.32 | 3.59 | 4.50 | 5.83 | 3.08 |
| SE | (0.60) | (0.99) | (0.69) | (0.66) | (0.80) | (0.74) | (0.69) | (0.61) | (1.24) | (0.48) |
| n | 83 | 90 | 122 | 71 | 62 | 126 | 120 | 113 | 99 | 95 |
| N | 1.98 | 2.94 | 2.31 | 1.69 | 1.53 | 2.00 | 1.67 | 2.08 | 2.71 | 1.40 |
|  | (3) Transition Into Coverage | | | | | | | | | |
| Pct | 7.04 | 7.60 | 6.94 | 5.76 | 7.52 | 6.64 | 8.33 | 7.97 | 6.76 | 5.87 |
| SE | (0.84) | (1.07) | (0.94) | (1.12) | (1.06) | (0.98) | (1.36) | (1.16) | (0.96) | (0.68) |
| n | 150 | 132 | 201 | 127 | 158 | 180 | 233 | 204 | 165 | 183 |
| N | 3.24 | 3.50 | 3.23 | 2.65 | 3.45 | 3.07 | 3.87 | 3.68 | 3.14 | 2.67 |
|  | (4) Transition Out of Coverage | | | | | | | | | |
| Pct | 4.84 | 6.59 | 4.08 | 5.22 | 4.22 | 4.67 | 4.93 | 3.57 | 2.48 | 2.55 |
| SE | (0.69) | (0.86) | (0.49) | (1.11) | (0.88) | (0.75) | (0.69) | (0.60) | (0.52) | (0.54) |
| n | 91 | 105 | 120 | 113 | 85 | 111 | 128 | 100 | 54 | 64 |
| N | 2.23 | 3.03 | 1.90 | 2.40 | 1.93 | 2.17 | 2.29 | 1.65 | 1.15 | 1.16 |
|  | (5) Temporary Coverage | | | | | | | | | |
| Pct | 0.98 | 1.35 | 1.71 | 1.13 | 0.66 | 1.25 | 0.81 | 0.44 | 0.81 | 0.96 |
| SE | (0.26) | (0.44) | (0.55) | (0.38) | (0.20) | (0.28) | (0.23) | (0.14) | (0.23) | (0.25) |
| n | 22 | 23 | 40 | 24 | 19 | 32 | 28 | 22 | 24 | 29 |
| N | .45 | .62 | .80 | .52 | .30 | .58 | .38 | .20 | .38 | .44 |
|  | (6) Repeatedly Uninsured | | | | | | | | | |
| Pct | 0.82 | 0.94 | 0.82 | 1.38 | 1.35 | 0.83 | 0.88 | 1.25 | 1.36 | 1.16 |
| SE | (0.21) | (0.25) | (0.22) | (0.31) | (0.43) | (0.21) | (0.24) | (0.36) | (0.33) | (0.33) |
| n | 24 | 26 | 32 | 29 | 24 | 31 | 26 | 37 | 31 | 35 |
| N | .38 | .43 | .38 | .63 | .62 | .39 | .41 | .58 | .63 | .53 |
|  | (7) Always Uninsured | | | | | | | | | |
| Pct | 7.95 | 7.81 | 6.83 | 9.37 | 8.53 | 7.55 | 7.92 | 5.86 | 4.39 | 4.88 |
| SE | (0.89) | (1.13) | (0.69) | (1.06) | (0.93) | (0.92) | (0.84) | (1.04) | (0.66) | (0.87) |
| n | 165 | 134 | 215 | 216 | 224 | 249 | 298 | 193 | 123 | 153 |
| N | 3.66 | 3.59 | 3.18 | 4.31 | 3.92 | 3.50 | 3.68 | 2.71 | 2.04 | 2.22 |
|  | (8) Any Insurance Interruption, i.e. Patterns Two to Seven | | | | | | | | | |
| Pct | 25.92 | 30.69 | 25.34 | 26.54 | 25.61 | 25.27 | 26.46 | 23.59 | 21.63 | 18.50 |
| SE | (1.36) | (1.77) | (1.92) | (2.12) | (2.23) | (2.11) | (1.67) | (2.20) | (1.69) | (1.59) |
| n | 535 | 510 | 730 | 580 | 572 | 729 | 833 | 669 | 496 | 559 |
| N | 11.94 | 14.11 | 11.79 | 12.22 | 11.75 | 11.71 | 12.30 | 10.90 | 10.06 | 8.41 |
| ^a^Percentage of a pattern in the total seven patterns; ^b^Standard Error of the percentage;  ^c^Number of observations in the sample; ^d^Sample-weighted population in millions for a pattern. | | | | | | | | | | |

| **Table A13 Distributions of Insurance Patterns from 2006 to 2016, Midwestern Region** | | | | | | | | | | |
| --- | --- | --- | --- | --- | --- | --- | --- | --- | --- | --- |
| Year | 2006-2007 | 2007-2008 | 2008-2009 | 2009-2010 | 2010-2011 | 2011-2012 | 2012-2013 | 2013-2014 | 2014-2015 | 2015-2016 |
|  | (1) Always Insured | | | | | | | | | |
| Pct^a^ | 75.54 | 68.79 | 68.48 | 72.04 | 71.34 | 75.53 | 69.93 | 73.09 | 75.86 | 76.15 |
| SE^b^ | (1.26) | (2.05) | (1.65) | (1.45) | (1.21) | (1.54) | (1.98) | (1.72) | (1.60) | (1.41) |
| n^c^ | 2039 | 1435 | 1957 | 1995 | 1697 | 2127 | 1816 | 1759 | 1704 | 1918 |
| N^d^ | 41.89 | 38.45 | 37.87 | 40.47 | 39.55 | 41.71 | 39.40 | 41.29 | 42.41 | 42.39 |
|  | (2) Single Gap | | | | | | | | | |
| Pct | 3.75 | 5.72 | 6.42 | 5.25 | 7.13 | 4.21 | 5.52 | 6.10 | 4.70 | 6.08 |
| SE | (0.48) | (0.76) | (0.88) | (0.81) | (0.73) | (0.51) | (0.83) | (0.93) | (0.81) | (0.71) |
| n | 107 | 135 | 174 | 135 | 161 | 154 | 139 | 159 | 118 | 176 |
| N | 2.08 | 3.20 | 3.55 | 2.95 | 3.95 | 2.32 | 3.11 | 3.45 | 2.63 | 3.39 |
|  | (3) Transition Into Coverage | | | | | | | | | |
| Pct | 5.28 | 8.47 | 6.69 | 4.92 | 5.42 | 5.27 | 6.23 | 7.56 | 7.66 | 5.39 |
| SE | (0.66) | (0.84) | (0.68) | (0.70) | (0.58) | (0.58) | (0.75) | (0.79) | (0.93) | (0.58) |
| n | 151 | 187 | 231 | 121 | 149 | 170 | 178 | 186 | 220 | 151 |
| N | 2.93 | 4.73 | 3.70 | 2.77 | 3.00 | 2.91 | 3.51 | 4.27 | 4.28 | 3.00 |
|  | (4) Transition Out of Coverage | | | | | | | | | |
| Pct | 5.61 | 4.39 | 6.15 | 4.56 | 4.72 | 5.47 | 5.22 | 3.23 | 3.71 | 3.85 |
| SE | (0.71) | (0.53) | (0.63) | (0.48) | (0.56) | (0.69) | (0.62) | (0.49) | (0.59) | (0.55) |
| n | 154 | 114 | 171 | 146 | 148 | 173 | 147 | 87 | 101 | 110 |
| N | 3.11 | 2.45 | 3.40 | 2.56 | 2.62 | 3.02 | 2.94 | 1.82 | 2.08 | 2.14 |
|  | (5) Temporary Coverage | | | | | | | | | |
| Pct | 0.75 | 1.17 | 1.08 | 1.13 | 0.90 | 0.99 | 1.82 | 0.86 | 1.27 | 1.01 |
| SE | (0.23) | (0.24) | (0.25) | (0.22) | (0.25) | (0.25) | (0.67) | (0.16) | (0.36) | (0.20) |
| n | 27 | 26 | 44 | 38 | 27 | 37 | 42 | 24 | 40 | 31 |
| N | .41 | .65 | .60 | .63 | .50 | .55 | 1.03 | .49 | .71 | .56 |
|  | (6) Repeatedly Uninsured | | | | | | | | | |
| Pct | 1.33 | 2.01 | 1.45 | 1.55 | 1.26 | 1.51 | 1.46 | 1.64 | 1.31 | 2.07 |
| SE | (0.37) | (0.40) | (0.25) | (0.42) | (0.23) | (0.31) | (0.29) | (0.34) | (0.25) | (0.56) |
| n | 37 | 46 | 44 | 46 | 40 | 46 | 60 | 54 | 45 | 52 |
| N | .74 | 1.13 | .80 | .87 | .70 | .83 | .83 | .93 | .73 | 1.15 |
|  | (7) Always Uninsured | | | | | | | | | |
| Pct | 7.73 | 9.45 | 9.72 | 10.55 | 9.23 | 7.03 | 9.82 | 7.52 | 5.49 | 5.46 |
| SE | (0.76) | (1.38) | (0.97) | (0.86) | (0.82) | (0.76) | (1.20) | (1.07) | (0.87) | (0.78) |
| n | 209 | 220 | 329 | 322 | 275 | 263 | 314 | 235 | 156 | 177 |
| N | 4.29 | 5.28 | 5.38 | 5.93 | 5.12 | 3.88 | 5.53 | 4.25 | 3.07 | 3.04 |
|  | (8) Any Insurance Interruption, i.e. Patterns Two to Seven | | | | | | | | | |
| Pct | 24.46 | 31.21 | 31.52 | 27.96 | 28.66 | 24.47 | 30.07 | 26.91 | 24.14 | 23.85 |
| SE | (1.26) | (2.05) | (1.65) | (1.45) | (1.21) | (1.54) | (1.98) | (1.72) | (1.60) | (1.41) |
| n | 685 | 728 | 993 | 808 | 800 | 843 | 880 | 745 | 680 | 697 |
| N | 13.56 | 17.45 | 17.43 | 15.71 | 15.89 | 13.52 | 16.94 | 15.20 | 13.49 | 13.28 |
| ^a^Percentage of a pattern in the total seven patterns; ^b^Standard Error of the percentage;  ^c^Number of observations in the sample; ^d^Sample-weighted population in millions for a pattern. | | | | | | | | | | |

| **Table A14 Distributions of Insurance Patterns from 2006 to 2016, Southern Region** | | | | | | | | | | |
| --- | --- | --- | --- | --- | --- | --- | --- | --- | --- | --- |
| Year | 2006-2007 | 2007-2008 | 2008-2009 | 2009-2010 | 2010-2011 | 2011-2012 | 2012-2013 | 2013-2014 | 2014-2015 | 2015-2016 |
|  | (1) Always Insured | | | | | | | | | |
| Pct^a^ | 63.92 | 61.84 | 61.52 | 66.04 | 66.92 | 63.09 | 63.86 | 65.98 | 66.71 | 70.01 |
| SE^b^ | (1.24) | (1.68) | (1.31) | (1.47) | (1.38) | (1.55) | (1.59) | (1.47) | (1.49) | (1.59) |
| n^c^ | 3027 | 2229 | 3358 | 3039 | 2688 | 3378 | 3214 | 3004 | 3074 | 3429 |
| N^d^ | 59.16 | 58.12 | 58.66 | 63.47 | 65.22 | 60.95 | 62.38 | 65.07 | 66.16 | 69.86 |
|  | (2) Single Gap | | | | | | | | | |
| Pct | 5.18 | 4.43 | 4.72 | 4.21 | 4.21 | 4.75 | 3.92 | 4.79 | 5.19 | 5.80 |
| SE | (0.46) | (0.45) | (0.49) | (0.43) | (0.51) | (0.55) | (0.58) | (0.57) | (0.59) | (1.00) |
| n | 300 | 178 | 303 | 240 | 203 | 275 | 229 | 267 | 274 | 246 |
| N | 4.79 | 4.16 | 4.50 | 4.05 | 4.10 | 4.59 | 3.83 | 4.73 | 5.14 | 5.79 |
|  | (3) Transition Into Coverage | | | | | | | | | |
| Pct | 6.88 | 8.62 | 7.40 | 5.93 | 7.64 | 7.03 | 7.92 | 9.01 | 8.99 | 6.69 |
| SE | (0.44) | (0.77) | (0.66) | (0.43) | (0.61) | (0.53) | (0.64) | (0.53) | (0.69) | (0.54) |
| n | 400 | 345 | 479 | 363 | 392 | 477 | 490 | 547 | 519 | 422 |
| N | 6.36 | 8.10 | 7.05 | 5.70 | 7.44 | 6.79 | 7.73 | 8.88 | 8.92 | 6.67 |
|  | (4) Transition Out of Coverage | | | | | | | | | |
| Pct | 6.80 | 5.73 | 6.46 | 6.31 | 5.08 | 5.70 | 6.27 | 4.17 | 5.07 | 4.32 |
| SE | (0.62) | (0.51) | (0.54) | (0.58) | (0.40) | (0.52) | (0.58) | (0.42) | (0.62) | (0.39) |
| n | 385 | 267 | 417 | 338 | 266 | 375 | 391 | 268 | 275 | 287 |
| N | 6.30 | 5.38 | 6.16 | 6.07 | 4.95 | 5.51 | 6.13 | 4.11 | 5.03 | 4.31 |
|  | (5) Temporary Coverage | | | | | | | | | |
| Pct | 1.64 | 1.29 | 1.96 | 1.31 | 1.16 | 2.05 | 1.29 | 1.50 | 1.75 | 1.79 |
| SE | (0.23) | (0.26) | (0.28) | (0.18) | (0.13) | (0.38) | (0.24) | (0.23) | (0.26) | (0.27) |
| n | 112 | 61 | 135 | 97 | 76 | 124 | 86 | 95 | 107 | 121 |
| N | 1.52 | 1.21 | 1.87 | 1.26 | 1.13 | 1.98 | 1.26 | 1.48 | 1.74 | 1.79 |
|  | (6) Repeatedly Uninsured | | | | | | | | | |
| Pct | 1.55 | 1.69 | 2.09 | 1.58 | 1.63 | 1.52 | 1.50 | 1.81 | 1.78 | 1.59 |
| SE | (0.24) | (0.27) | (0.29) | (0.24) | (0.26) | (0.22) | (0.29) | (0.27) | (0.27) | (0.24) |
| n | 121 | 69 | 128 | 100 | 95 | 99 | 103 | 116 | 107 | 97 |
| N | 1.43 | 1.59 | 2.00 | 1.51 | 1.59 | 1.47 | 1.46 | 1.79 | 1.76 | 1.58 |
|  | (7) Always Uninsured | | | | | | | | | |
| Pct | 14.03 | 16.40 | 15.84 | 14.62 | 13.35 | 15.86 | 15.24 | 12.74 | 10.51 | 9.79 |
| SE | (0.82) | (1.14) | (0.83) | (1.24) | (0.91) | (0.92) | (1.25) | (0.89) | (0.82) | (0.78) |
| n | 923 | 759 | 1126 | 916 | 859 | 1244 | 1131 | 905 | 769 | 772 |
| N | 12.99 | 15.41 | 15.10 | 14.05 | 13.01 | 15.32 | 14.89 | 12.56 | 10.42 | 9.77 |
|  | (8) Any Insurance Interruption, i.e. Patterns Two to Seven | | | | | | | | | |
| Pct | 36.08 | 38.16 | 38.48 | 33.96 | 33.08 | 36.91 | 36.14 | 34.02 | 33.29 | 29.99 |
| SE | (1.24) | (1.68) | (1.31) | (1.47) | (1.38) | (1.55) | (1.59) | (1.47) | (1.49) | (1.59) |
| n | 2241 | 1679 | 2588 | 2054 | 1891 | 2594 | 2430 | 2198 | 2051 | 1945 |
| N | 33.39 | 35.87 | 36.69 | 32.63 | 32.24 | 35.66 | 35.30 | 33.55 | 33.01 | 29.92 |
| ^a^Percentage of a pattern in the total seven patterns; ^b^Standard Error of the percentage;  ^c^Number of observations in the sample; ^d^Sample-weighted population in millions for a pattern. | | | | | | | | | | |

| **Table A15 Distributions of Insurance Patterns from 2006 to 2016, Western Region** | | | | | | | | | | |
| --- | --- | --- | --- | --- | --- | --- | --- | --- | --- | --- |
| Year | 2006-2007 | 2007-2008 | 2008-2009 | 2009-2010 | 2010-2011 | 2011-2012 | 2012-2013 | 2013-2014 | 2014-2015 | 2015-2016 |
|  | (1) Always Insured | | | | | | | | | |
| Pct^a^ | 64.06 | 66.88 | 61.73 | 64.56 | 65.20 | 67.35 | 65.44 | 64.92 | 71.13 | 74.34 |
| SE^b^ | (1.48) | (1.66) | (1.76) | (1.52) | (1.44) | (1.27) | (1.37) | (1.78) | (1.45) | (1.17) |
| n^c^ | 2327 | 1795 | 2561 | 2335 | 1979 | 2680 | 2653 | 2555 | 2642 | 2759 |
| N^d^ | 38.49 | 40.44 | 37.63 | 39.22 | 40.14 | 42.34 | 40.50 | 40.81 | 44.96 | 47.21 |
|  | (2) Single Gap | | | | | | | | | |
| Pct | 5.29 | 4.52 | 5.05 | 5.12 | 4.22 | 3.98 | 4.65 | 7.71 | 5.45 | 5.42 |
| SE | (0.66) | (0.54) | (0.81) | (0.65) | (0.57) | (0.40) | (0.60) | (1.10) | (0.79) | (0.64) |
| n | 229 | 140 | 186 | 207 | 146 | 164 | 211 | 272 | 217 | 218 |
| N | 3.18 | 2.74 | 3.08 | 3.11 | 2.60 | 2.50 | 2.88 | 4.85 | 3.44 | 3.45 |
|  | (3) Transition Into Coverage | | | | | | | | | |
| Pct | 8.89 | 8.90 | 7.60 | 7.32 | 7.56 | 7.26 | 7.47 | 10.92 | 10.48 | 7.33 |
| SE | (0.73) | (1.02) | (0.70) | (0.79) | (0.66) | (0.57) | (0.61) | (0.82) | (0.96) | (0.65) |
| n | 356 | 249 | 377 | 306 | 247 | 339 | 356 | 506 | 487 | 350 |
| N | 5.34 | 5.38 | 4.63 | 4.45 | 4.65 | 4.57 | 4.62 | 6.86 | 6.62 | 4.65 |
|  | (4) Transition Out of Coverage | | | | | | | | | |
| Pct | 6.45 | 5.62 | 6.32 | 6.58 | 5.92 | 5.22 | 5.08 | 3.73 | 3.62 | 4.77 |
| SE | (0.83) | (0.58) | (0.60) | (0.70) | (0.60) | (0.47) | (0.44) | (0.39) | (0.53) | (0.56) |
| n | 274 | 168 | 295 | 243 | 203 | 235 | 261 | 171 | 146 | 178 |
| N | 3.88 | 3.40 | 3.85 | 4.00 | 3.64 | 3.28 | 3.14 | 2.34 | 2.29 | 3.03 |
|  | (5) Temporary Coverage | | | | | | | | | |
| Pct | 1.02 | 2.05 | 1.68 | 1.03 | 1.27 | 1.18 | 1.54 | 0.94 | 1.18 | 1.50 |
| SE | (0.22) | (0.33) | (0.28) | (0.16) | (0.20) | (0.20) | (0.24) | (0.20) | (0.26) | (0.25) |
| n | 51 | 58 | 82 | 54 | 48 | 59 | 71 | 49 | 52 | 69 |
| N | .61 | 1.24 | 1.02 | .63 | .78 | .74 | .95 | .59 | .75 | .95 |
|  | (6) Repeatedly Uninsured | | | | | | | | | |
| Pct | 1.75 | 2.01 | 2.44 | 1.73 | 1.68 | 1.36 | 1.17 | 1.78 | 1.52 | 1.70 |
| SE | (0.44) | (0.34) | (0.42) | (0.33) | (0.41) | (0.36) | (0.23) | (0.36) | (0.25) | (0.32) |
| n | 85 | 54 | 111 | 61 | 53 | 63 | 60 | 75 | 67 | 76 |
| N | 1.05 | 1.22 | 1.49 | 1.05 | 1.04 | .86 | .72 | 1.12 | .96 | 1.08 |
|  | (7) Always Uninsured | | | | | | | | | |
| Pct | 12.54 | 10.01 | 15.17 | 13.66 | 14.16 | 13.65 | 14.65 | 9.99 | 6.63 | 4.93 |
| SE | (0.96) | (0.73) | (1.41) | (0.93) | (0.98) | (1.04) | (0.95) | (1.00) | (0.64) | (0.48) |
| n | 610 | 323 | 765 | 620 | 568 | 672 | 818 | 542 | 361 | 283 |
| N | 7.53 | 6.05 | 9.25 | 8.30 | 8.72 | 8.58 | 9.07 | 6.28 | 4.19 | 3.13 |
|  | (8) Any Insurance Interruption, i.e. Patterns Two to Seven | | | | | | | | | |
| Pct | 35.94 | 33.12 | 38.27 | 35.44 | 34.80 | 32.65 | 34.56 | 35.08 | 28.87 | 25.66 |
| SE | (1.48) | (1.66) | (1.76) | (1.52) | (1.44) | (1.27) | (1.37) | (1.78) | (1.45) | (1.17) |
| n | 1605 | 992 | 1816 | 1491 | 1265 | 1532 | 1777 | 1615 | 1330 | 1174 |
| N | 21.59 | 20.03 | 23.32 | 21.53 | 21.43 | 20.52 | 21.39 | 22.05 | 18.25 | 16.30 |
| ^a^Percentage of a pattern in the total seven patterns; ^b^Standard Error of the percentage;  ^c^Number of observations in the sample; ^d^Sample-weighted population in millions for a pattern. | | | | | | | | | | |

| **Table A16 Distributions of Insurance Patterns from 2006 to 2016, Respondents with Priority Conditions** | | | | | | | | | | |
| --- | --- | --- | --- | --- | --- | --- | --- | --- | --- | --- |
| Year | 2006-2007 | 2007-2008 | 2008-2009 | 2009-2010 | 2010-2011 | 2011-2012 | 2012-2013 | 2013-2014 | 2014-2015 | 2015-2016 |
|  | (1) Always Insured | | | | | | | | | |
| Pct^a^ | 70.81 | 68.08 | 67.27 | 70.74 | 70.25 | 69.99 | 68.39 | 68.99 | 72.61 | 74.67 |
| SE^b^ | (0.80) | (0.98) | (0.95) | (0.92) | (0.84) | (0.89) | (0.92) | (0.96) | (0.88) | (0.88) |
| n^c^ | 4281 | 3556 | 4901 | 4668 | 3936 | 5023 | 4831 | 4636 | 4589 | 5110 |
| N^d^ | 88.52 | 94.65 | 96.47 | 99.69 | 99.28 | 97.73 | 97.57 | 102.48 | 107.42 | 109.30 |
|  | (2) Single Gap | | | | | | | | | |
| Pct | 4.17 | 4.96 | 4.53 | 4.33 | 4.31 | 3.77 | 4.25 | 5.71 | 4.87 | 5.42 |
| SE | (0.34) | (0.38) | (0.38) | (0.38) | (0.32) | (0.31) | (0.38) | (0.45) | (0.41) | (0.49) |
| n | 275 | 262 | 348 | 297 | 270 | 328 | 351 | 429 | 349 | 390 |
| N | 5.21 | 6.89 | 6.50 | 6.10 | 6.09 | 5.27 | 6.06 | 8.48 | 7.21 | 7.94 |
|  | (3) Transition Into Coverage | | | | | | | | | |
| Pct | 6.59 | 7.67 | 7.01 | 5.48 | 7.09 | 6.72 | 7.45 | 9.83 | 8.96 | 6.45 |
| SE | (0.45) | (0.48) | (0.44) | (0.37) | (0.43) | (0.37) | (0.50) | (0.49) | (0.54) | (0.38) |
| n | 456 | 424 | 617 | 435 | 465 | 588 | 615 | 826 | 758 | 582 |
| N | 8.23 | 10.66 | 10.05 | 7.72 | 10.02 | 9.39 | 10.63 | 14.61 | 13.26 | 9.45 |
|  | (4) Transition Out of Coverage | | | | | | | | | |
| Pct | 5.79 | 5.38 | 6.08 | 5.69 | 5.08 | 5.18 | 5.17 | 3.73 | 3.80 | 3.67 |
| SE | (0.43) | (0.34) | (0.37) | (0.43) | (0.41) | (0.37) | (0.33) | (0.31) | (0.36) | (0.34) |
| n | 383 | 319 | 521 | 436 | 351 | 427 | 438 | 310 | 272 | 315 |
| N | 7.24 | 7.49 | 8.71 | 8.02 | 7.18 | 7.24 | 7.37 | 5.55 | 5.62 | 5.37 |
|  | (5) Temporary Coverage | | | | | | | | | |
| Pct | 1.15 | 1.28 | 1.26 | 1.12 | 0.93 | 1.41 | 1.10 | 1.03 | 1.35 | 1.53 |
| SE | (0.16) | (0.18) | (0.15) | (0.15) | (0.11) | (0.17) | (0.15) | (0.14) | (0.17) | (0.18) |
| n | 84 | 79 | 130 | 93 | 79 | 131 | 108 | 89 | 119 | 144 |
| N | 1.44 | 1.78 | 1.81 | 1.57 | 1.31 | 1.97 | 1.57 | 1.53 | 1.99 | 2.24 |
|  | (6) Repeatedly Uninsured | | | | | | | | | |
| Pct | 1.21 | 1.64 | 1.64 | 1.40 | 1.32 | 1.48 | 1.34 | 1.65 | 1.72 | 1.75 |
| SE | (0.18) | (0.19) | (0.21) | (0.18) | (0.17) | (0.17) | (0.18) | (0.21) | (0.21) | (0.21) |
| n | 96 | 88 | 141 | 112 | 99 | 128 | 122 | 139 | 133 | 147 |
| N | 1.51 | 2.28 | 2.36 | 1.97 | 1.87 | 2.07 | 1.92 | 2.45 | 2.54 | 2.57 |
|  | (7) Always Uninsured | | | | | | | | | |
| Pct | 10.28 | 10.98 | 12.21 | 11.25 | 11.02 | 11.43 | 12.30 | 9.06 | 6.69 | 6.50 |
| SE | (0.54) | (0.55) | (0.57) | (0.57) | (0.51) | (0.54) | (0.64) | (0.56) | (0.49) | (0.45) |
| n | 820 | 687 | 1180 | 994 | 921 | 1141 | 1231 | 896 | 667 | 702 |
| N | 12.85 | 15.27 | 17.51 | 15.86 | 15.57 | 15.97 | 17.54 | 13.45 | 9.89 | 9.51 |
|  | (8) Any Insurance Interruption, i.e. Patterns Two to Seven | | | | | | | | | |
| Pct | 29.19 | 31.92 | 32.73 | 29.26 | 29.75 | 30.01 | 31.61 | 31.01 | 27.39 | 25.33 |
| SE | (0.80) | (0.98) | (0.95) | (0.92) | (0.84) | (0.89) | (0.92) | (0.96) | (0.88) | (0.88) |
| n | 2114 | 1859 | 2937 | 2367 | 2185 | 2743 | 2865 | 2689 | 2298 | 2280 |
| N | 36.48 | 44.37 | 46.93 | 41.24 | 42.05 | 41.91 | 45.09 | 46.07 | 40.52 | 37.07 |
| ^a^Percentage of a pattern in the total seven patterns; ^b^Standard Error of the percentage;  ^c^Number of observations in the sample; ^d^Sample-weighted population in millions for a pattern. | | | | | | | | | | |

| **Table A17 Distributions of Insurance Patterns from 2006 to 2016,**  **Respondents without Priority Conditions** | | | | | | | | | | |
| --- | --- | --- | --- | --- | --- | --- | --- | --- | --- | --- |
| Year | 2006-2007 | 2007-2008 | 2008-2009 | 2009-2010 | 2010-2011 | 2011-2012 | 2012-2013 | 2013-2014 | 2014-2015 | 2015-2016 |
|  | (1) Always Insured | | | | | | | | | |
| Pct^a^ | 65.95 | 63.27 | 63.13 | 65.43 | 67.02 | 67.45 | 65.89 | 69.17 | 70.65 | 73.88 |
| SE^b^ | (0.95) | (1.38) | (1.07) | (1.13) | (1.05) | (1.08) | (1.13) | (1.20) | (1.15) | (1.08) |
| n^c^ | 4487 | 3032 | 4601 | 4078 | 3776 | 4868 | 4572 | 4343 | 4243 | 4639 |
| N^d^ | 85.16 | 74.23 | 72.42 | 77.28 | 79.76 | 81.87 | 78.90 | 79.94 | 82.52 | 87.21 |
|  | (2) Single Gap | | | | | | | | | |
| Pct | 5.28 | 5.24 | 6.06 | 4.83 | 5.11 | 5.06 | 4.53 | 5.72 | 5.75 | 5.15 |
| SE | (0.38) | (0.52) | (0.50) | (0.41) | (0.47) | (0.38) | (0.45) | (0.56) | (0.63) | (0.55) |
| n | 444 | 281 | 437 | 356 | 302 | 391 | 348 | 382 | 359 | 345 |
| N | 6.82 | 6.15 | 6.95 | 5.70 | 6.09 | 6.15 | 5.42 | 6.62 | 6.71 | 6.08 |
|  | (3) Transition Into Coverage | | | | | | | | | |
| Pct | 7.47 | 9.42 | 7.46 | 6.64 | 7.16 | 6.55 | 7.61 | 7.87 | 8.30 | 6.39 |
| SE | (0.44) | (0.66) | (0.45) | (0.53) | (0.53) | (0.46) | (0.56) | (0.57) | (0.58) | (0.43) |
| n | 601 | 489 | 671 | 482 | 481 | 578 | 642 | 617 | 633 | 524 |
| N | 9.64 | 11.05 | 8.56 | 7.84 | 8.52 | 7.95 | 9.11 | 9.09 | 9.70 | 7.55 |
|  | (4) Transition Out of Coverage | | | | | | | | | |
| Pct | 6.39 | 5.78 | 5.75 | 5.93 | 5.02 | 5.55 | 5.95 | 3.79 | 4.22 | 4.47 |
| SE | (0.46) | (0.50) | (0.44) | (0.46) | (0.41) | (0.41) | (0.48) | (0.31) | (0.42) | (0.34) |
| n | 520 | 335 | 482 | 404 | 351 | 467 | 489 | 316 | 304 | 324 |
| N | 8.25 | 6.78 | 6.60 | 7.01 | 5.97 | 6.74 | 7.13 | 4.38 | 4.93 | 5.28 |
|  | (5) Temporary Coverage | | | | | | | | | |
| Pct | 1.20 | 1.66 | 2.16 | 1.24 | 1.18 | 1.55 | 1.70 | 1.07 | 1.35 | 1.27 |
| SE | (0.16) | (0.24) | (0.33) | (0.14) | (0.15) | (0.26) | (0.29) | (0.17) | (0.20) | (0.16) |
| n | 128 | 89 | 171 | 120 | 91 | 121 | 119 | 101 | 104 | 106 |
| N | 1.56 | 1.95 | 2.48 | 1.46 | 1.40 | 1.88 | 2.04 | 1.23 | 1.58 | 1.50 |
|  | (6) Repeatedly Uninsured | | | | | | | | | |
| Pct | 1.62 | 1.78 | 2.02 | 1.78 | 1.75 | 1.21 | 1.26 | 1.70 | 1.32 | 1.51 |
| SE | (0.22) | (0.25) | (0.21) | (0.26) | (0.29) | (0.18) | (0.21) | (0.21) | (0.15) | (0.22) |
| n | 171 | 107 | 174 | 124 | 113 | 111 | 127 | 143 | 117 | 113 |
| N | 2.09 | 2.08 | 2.31 | 2.10 | 2.08 | 1.47 | 1.50 | 1.96 | 1.55 | 1.78 |
|  | (7) Always Uninsured | | | | | | | | | |
| Pct | 12.09 | 12.85 | 13.42 | 14.15 | 12.77 | 12.62 | 13.06 | 10.68 | 8.41 | 7.33 |
| SE | (0.66) | (1.03) | (0.73) | (0.79) | (0.70) | (0.65) | (0.73) | (0.71) | (0.52) | (0.52) |
| n | 1087 | 749 | 1255 | 1079 | 1005 | 1287 | 1330 | 979 | 742 | 683 |
| N | 15.62 | 15.07 | 15.39 | 16.72 | 15.19 | 15.31 | 15.63 | 12.35 | 9.82 | 8.65 |
|  | (8) Any Insurance Interruption, i.e. Patterns Two to Seven | | | | | | | | | |
| Pct | 34.05 | 36.73 | 36.87 | 34.57 | 32.98 | 32.55 | 34.11 | 30.83 | 29.35 | 26.12 |
| SE | (0.95) | (1.38) | (1.07) | (1.13) | (1.05) | (1.08) | (1.13) | (1.20) | (1.15) | (1.08) |
| n | 2951 | 2050 | 3190 | 2565 | 2343 | 2955 | 3055 | 2538 | 2259 | 2095 |
| N | 43.97 | 43.08 | 42.29 | 40.83 | 39.26 | 39.50 | 40.84 | 35.63 | 34.29 | 30.84 |
| ^a^Percentage of a pattern in the total seven patterns; ^b^Standard Error of the percentage;  ^c^Number of observations in the sample; ^d^Sample-weighted population in millions for a pattern. | | | | | | | | | | |
