# Supplementary material for: Insurance patterns and instability from 2006 to 2016
Source: BMC Health Serv Res. 2020 Apr 21;20:334. doi: 10.1186/s12913-020-05226-1 (PMC7171789; doi:10.1186/s12913-020-05226-1)
Supplement: Supplementary file 2 — Additional file 2. Appendix B: robustness tests using logit models for dichotomous dependent variables, Poisson models for count variables and log-linear models for continuous dependent variables [file 12913_2020_5226_MOESM2_ESM.docx]

**Appendix B**

In the robustness tests, we use logit models for dichotomous dependent variables, Poisson models for count variables and log-linear models for continuous dependent variables. Table B1 lists odds ratios from logit models, incidence rate ratios from Poisson models, and coefficients from log-linear models. To be concise, we only report the coefficients for instability patterns in each of the 16 models, where “always insured” is the comparison group. Full results are available from the authors upon request.

In Table B2, we created a dummy variable that equaled one if there were transitions between private and public insurance. We then included it as an additional independent variable in all models. As shown in this table, the changes in the coefficients for insurance patterns were small and comparable to the results in Table 4 in the manuscript. Therefore, our conclusions remain the same.

In Table B3, instead of using dummy variables to capture different types of insurance interruptions, we used the number of months a person was uninsured. The coefficient for this variable can be directly interpreted as the dose response, i.e., how the spell of non-coverage affects the health status and other outcomes.

| **Table B1. Relationships of Insurance Patterns with Health Care Usage, Status and Outcomes** | | | | | | | | |
| --- | --- | --- | --- | --- | --- | --- | --- | --- |
|  | Lack of Access^a^ | Any Prev. Care^a^ | # of Prev. Care^b^ | Smoking^a^ | Physical Activity^a^ | Pcvd. Poor Hlth^a^ | Pcvd. Poor Mental Hlth^a^ | Owt. or Obese^a^ |
| Single Gap | 2.19*** | 0.90 | 0.99** | 1.29*** | 1.09* | 0.86** | 0.72*** | 0.99 |
|  | (0.10) | (0.11) | (0.01) | (0.07) | (0.05) | (0.06) | (0.07) | (0.05) |
|  |  |  |  |  |  |  |  |  |
| Transition Into | 2.76*** | 0.43*** | 0.92*** | 1.36*** | 1.12*** | 0.86*** | 0.77*** | 0.92** |
|  | (0.10) | (0.04) | (0.00) | (0.06) | (0.04) | (0.05) | (0.05) | (0.03) |
|  |  |  |  |  |  |  |  |  |
| Transition Out | 2.66*** | 0.38*** | 0.92*** | 1.44*** | 1.09* | 0.66*** | 0.62*** | 0.96 |
|  | (0.11) | (0.03) | (0.01) | (0.07) | (0.05) | (0.05) | (0.06) | (0.04) |
|  |  |  |  |  |  |  |  |  |
| Temporary Coverage | 4.09*** | 0.31*** | 0.89*** | 1.52*** | 0.95 | 0.71*** | 0.54*** | 0.90 |
|  | (0.31) | (0.04) | (0.01) | (0.11) | (0.06) | (0.08) | (0.09) | (0.06) |
|  |  |  |  |  |  |  |  |  |
| Repeatedly Uninsured | 3.33*** | 0.48*** | 0.94*** | 1.62*** | 1.08 | 0.77** | 0.68*** | 1.17** |
|  | (0.24) | (0.08) | (0.01) | (0.13) | (0.08) | (0.08) | (0.09) | (0.09) |
|  |  |  |  |  |  |  |  |  |
| Always Uninsured | 5.19*** | 0.14*** | 0.71*** | 1.46*** | 1.11*** | 0.67*** | 0.59*** | 0.81*** |
|  | (0.21) | (0.01) | (0.01) | (0.05) | (0.04) | (0.03) | (0.04) | (0.02) |
|  |  |  |  |  |  |  |  |  |
| # of obs. | 123324 | 123148 | 123331 | 91064 | 94703 | 123331 | 123331 | 94181 |
|  |  |  |  |  |  |  |  |  |
|  | Kessler Index^c^ | PCS^c^ | MCS^c^ | BMI^c^ | Tot. Hlth Exp. ^c^ | Total Visits^b^ | Tot. Dental^b^ | Tot. Rx^b^ |
| Single Gap | 0.04* | 0.03*** | -0.00 | -0.01 | -0.25 | 0.60*** | 0.87 | 0.61* |
|  | (0.02) | (0.00) | (0.00) | (0.00) | (0.19) | (0.10) | (0.15) | (0.17) |
|  |  |  |  |  |  |  |  |  |
| Transition Into | 0.03 | 0.01*** | -0.00 | -0.02*** | -0.35** | 0.59*** | 0.61*** | 0.67* |
|  | (0.02) | (0.00) | (0.00) | (0.00) | (0.15) | (0.09) | (0.07) | (0.16) |
|  |  |  |  |  |  |  |  |  |
| Transition Out | 0.02 | 0.03*** | 0.00 | -0.01*** | -0.36*** | 0.65*** | 0.69*** | 0.81 |
|  | (0.02) | (0.00) | (0.00) | (0.00) | (0.10) | (0.10) | (0.09) | (0.13) |
|  |  |  |  |  |  |  |  |  |
| Temporary Coverage | 0.05 | 0.04*** | -0.01 | -0.01 | -0.64*** | 0.41*** | 0.49*** | 0.34*** |
|  | (0.03) | (0.01) | (0.01) | (0.01) | (0.21) | (0.07) | (0.10) | (0.06) |
|  |  |  |  |  |  |  |  |  |
| Repeatedly Uninsured | 0.10*** | 0.03*** | -0.01 | 0.01 | -0.14 | 0.59** | 0.53** | 0.44*** |
|  | (0.03) | (0.01) | (0.01) | (0.01) | (0.16) | (0.13) | (0.14) | (0.11) |
|  |  |  |  |  |  |  |  |  |
| Always Uninsured | 0.01 | 0.04*** | 0.00* | -0.03*** | -0.64*** | 0.61 | 0.46*** | 0.34*** |
|  | (0.01) | (0.00) | (0.00) | (0.00) | (0.15) | (0.21) | (0.07) | (0.08) |
|  |  |  |  |  |  |  |  |  |
| # of obs. | 69734 | 91230 | 91232 | 137660 | 3150 | 3706 | 3706 | 3706 |
| * p<0.10; ** p<0.05; *** p<0.01 Standard errors are in parentheses. | | | | | | | | |
| ^a^ Odds ratios from logit models. ^b^ Incidence rate ratios from Poisson models. ^c^ Coefficients from log-linear models. | | | | | | | | |
| We used MEPS’s longitudinal sample weights and stratum to account for the complex survey. | | | | | | | | |
| All models included respondents’ socioeconomic characteristics, demographics, general health status, priority conditions, panel indicators, and geographic indicators. Full results are available from the authors on request. | | | | | | | | |

| **Table B2. Relationships of Insurance Patterns and Transition with Health Care Usage, Status and Outcomes** | | | | | | | | |
| --- | --- | --- | --- | --- | --- | --- | --- | --- |
|  | **Lack of Access** | **Any Preventive Care** | **# of Preventive Care** | **Smoking** | **Physical Activity** | **Perceived Poor Health** | **Perceived Poor Mental Health** | **Overweight or Obese** |
| Single Gap | 0.17*** | -0.00 | -0.12*** | 0.04*** | 0.02* | -0.02*** | -0.02*** | -0.00 |
|  | (0.01) | (0.00) | (0.03) | (0.01) | (0.01) | (0.00) | (0.00) | (0.01) |
|  |  |  |  |  |  |  |  |  |
| Transition Into | 0.23*** | -0.02*** | -0.40*** | 0.05*** | 0.02*** | -0.01* | -0.01*** | -0.02** |
|  | (0.01) | (0.00) | (0.02) | (0.01) | (0.01) | (0.00) | (0.00) | (0.01) |
|  |  |  |  |  |  |  |  |  |
| Transition Out | 0.22*** | -0.03*** | -0.39*** | 0.06*** | 0.02* | -0.03*** | -0.02*** | -0.01 |
|  | (0.01) | (0.00) | (0.03) | (0.01) | (0.01) | (0.00) | (0.00) | (0.01) |
|  |  |  |  |  |  |  |  |  |
| Temporary Coverage | 0.31*** | -0.04*** | -0.54*** | 0.08*** | -0.01 | -0.03*** | -0.03*** | -0.02 |
|  | (0.01) | (0.01) | (0.06) | (0.01) | (0.01) | (0.01) | (0.01) | (0.01) |
|  |  |  |  |  |  |  |  |  |
| Repeatedly Uninsured | 0.27*** | -0.02*** | -0.33*** | 0.09*** | 0.02 | -0.02*** | -0.02*** | 0.03** |
|  | (0.02) | (0.01) | (0.04) | (0.02) | (0.01) | (0.01) | (0.01) | (0.01) |
|  |  |  |  |  |  |  |  |  |
| Always Uninsured | 0.36*** | -0.11*** | -1.31*** | 0.07*** | 0.02** | -0.03*** | -0.02*** | -0.04*** |
|  | (0.01) | (0.00) | (0.03) | (0.01) | (0.01) | (0.00) | (0.00) | (0.01) |
|  |  |  |  |  |  |  |  |  |
| Transitions priv. & pub. | 0.02* | -0.00 | 0.14*** | -0.00 | -0.01 | 0.02*** | 0.01*** | 0.02*** |
|  | (0.01) | (0.00) | (0.02) | (0.01) | (0.01) | (0.00) | (0.00) | (0.01) |
|  |  |  |  |  |  |  |  |  |
| # of obs. | 123324 | 123148 | 123331 | 91064 | 94703 | 123331 | 123331 | 94181 |
| R^2^ | 0.20 | 0.07 | 0.64 | 0.14 | 0.06 | 0.13 | 0.07 | 0.12 |
|  | **Kessler Index** | **Physical Comp. Summary** | **Mental Comp. Summary** | **BMI** | **Total Health Expenditure** | **# of Office Visits** | **# of Dental Visits** | **# of Prescriptions** |
| Single Gap | -0.03 | 1.30*** | -0.10 | -0.26* | -0.27 | -2.44*** | -0.27 | -2.80** |
|  | (0.08) | (0.16) | (0.20) | (0.13) | (1.21) | (0.71) | (0.34) | (1.20) |
|  |  |  |  |  |  |  |  |  |
| Transition Into | 0.07 | 0.41*** | -0.22 | -0.48*** | -0.66 | -2.14*** | -0.85*** | -2.26** |
|  | (0.06) | (0.12) | (0.14) | (0.10) | (0.62) | (0.57) | (0.17) | (1.01) |
|  |  |  |  |  |  |  |  |  |
| Transition Out | -0.10 | 1.13*** | -0.07 | -0.37*** | -1.57*** | -1.93*** | -0.68*** | -1.52** |
|  | (0.07) | (0.14) | (0.16) | (0.12) | (0.41) | (0.54) | (0.20) | (0.74) |
|  |  |  |  |  |  |  |  |  |
| Temporary Coverage | 0.12 | 1.08*** | -0.41 | -0.30 | -1.04* | -2.87*** | -1.07*** | -3.44*** |
|  | (0.14) | (0.25) | (0.30) | (0.20) | (0.61) | (0.58) | (0.22) | (0.78) |
|  |  |  |  |  |  |  |  |  |
| Repeatedly Uninsured | 0.24* | 1.54*** | -0.43 | 0.16 | -2.40*** | -2.07*** | -0.91*** | -4.05*** |
|  | (0.14) | (0.29) | (0.31) | (0.21) | (0.84) | (0.75) | (0.31) | (0.85) |
|  |  |  |  |  |  |  |  |  |
| Always Uninsured | -0.16*** | 1.27*** | 0.11 | -0.86*** | -1.83*** | -1.77 | -1.13*** | -3.02*** |
|  | (0.06) | (0.11) | (0.11) | (0.08) | (0.42) | (1.09) | (0.16) | (0.58) |
|  |  |  |  |  |  |  |  |  |
| Transitions priv. & pub. | 0.33*** | -1.89*** | -0.49*** | 0.20* | 0.75 | 0.61 | -0.25 | 2.73** |
|  | (0.08) | (0.18) | (0.16) | (0.12) | (0.68) | (0.66) | (0.22) | (1.20) |
|  |  |  |  |  |  |  |  |  |
| # of obs. | 90820 | 91230 | 91232 | 137660 | 3706 | 3706 | 3706 | 3706 |
| R^2^ | 0.13 | 0.25 | 0.10 | 0.11 | 0.22 | 0.19 | 0.10 | 0.26 |
| *p < 0.10; **p < 0.05; ***p < 0.01  Standard errors are reported in parentheses. MEPS’s longitudinal sample weights are applied in each regression. | | | | | | | | |
| All models included respondents’ socioeconomic characteristics, demographics, general health status, priority conditions, panel indicators, and geographic indicators. Please refer to “independent variables” section for a full list of the variables.  For conciseness, these coefficients are not reported here. Full results are available from the authors on request. | | | | | | | | |

| **Table B3. Relationships of Uninsured Months with Health Care Usage, Status and Outcomes** | | | | | | | | |
| --- | --- | --- | --- | --- | --- | --- | --- | --- |
|  | **Lack of Access** | **Any Preventive Care** | **# of Preventive Care** | **Smoking** | **Physical Activity** | **Perceived Poor Health** | **Perceived Poor Mental Health** | **Overweight or Obese** |
| # of month not insured | 0.0155*** | -0.00415*** | -0.0505*** | 0.00328*** | 0.000926*** | -0.00129*** | -0.00107*** | -0.00172*** |
|  | (51.36) | (-27.73) | (-48.82) | (12.75) | (3.19) | (-11.20) | (-11.08) | (-7.14) |
|  |  |  |  |  |  |  |  |  |
|  |  |  |  |  |  |  |  |  |
| # of obs. | 123324 | 123148 | 123331 | 91064 | 94703 | 123331 | 123331 | 94181 |
| R^2^ | 0.20 | 0.07 | 0.64 | 0.14 | 0.06 | 0.13 | 0.07 | 0.12 |
|  | **Kessler Index** | **Physical Comp. Summary** | **Mental Comp. Summary** | **BMI** | **Total Health Expenditure** | **# of Office Visits** | **# of Dental Visits** | **# of Prescriptions** |
| # of month not insured | -0.00567*** | 0.0579*** | 0.00309 | -0.0342*** | -0.0946*** | -0.113*** | -0.0562*** | -0.160*** |
|  | (-2.65) | (14.82) | (0.71) | (-10.01) | (-5.28) | (-3.23) | (-9.36) | (-6.31) |
|  |  |  |  |  |  |  |  |  |
|  |  |  |  |  |  |  |  |  |
| # of obs. | 90820 | 91230 | 91232 | 137660 | 3706 | 3706 | 3706 | 3706 |
| R^2^ | 0.13 | 0.25 | 0.10 | 0.11 | 0.22 | 0.19 | 0.10 | 0.26 |
| *p < 0.10; **p < 0.05; ***p < 0.01  Standard errors are reported in parentheses. MEPS’s longitudinal sample weights are applied in each regression. | | | | | | | | |
| All models included respondents’ socioeconomic characteristics, demographics, general health status, priority conditions, panel indicators, and geographic indicators. Please refer to “independent variables” section for a full list of the variables.  For conciseness, these coefficients are not reported here. Full results are available from the authors on request. | | | | | | | | |
